# Supplementary material for: Drosophila complement-like Mcr acts as a wound-induced inflammatory chemoattractant
Source: Curr Biol. 2025 Mar 18;35(7):1656–1664.e4. doi: 10.1016/j.cub.2025.02.036 (PMC12254109; doi:10.1016/j.cub.2025.02.036)
Supplement: Document S1. Figures S1–S5 and Tables S1 and S2 [file mmc1.pdf]

Current Biology, Volume *35*

Supplemental Information

Drosophila complement-like Mcr acts as a wound-induced inflammatory chemoattractant

Luigi Zechini, Henry Todd, Thibaut Sanchez, Daniel R. Tudor, Jennie S. Campbell, Edward Antonian, Stephen J. Jenkins, Christopher D. Lucas, Andrew J. Davidson, Jean van den Elsen, Linus J. Schumacher, Alessandro Scopelliti, and Will Wood

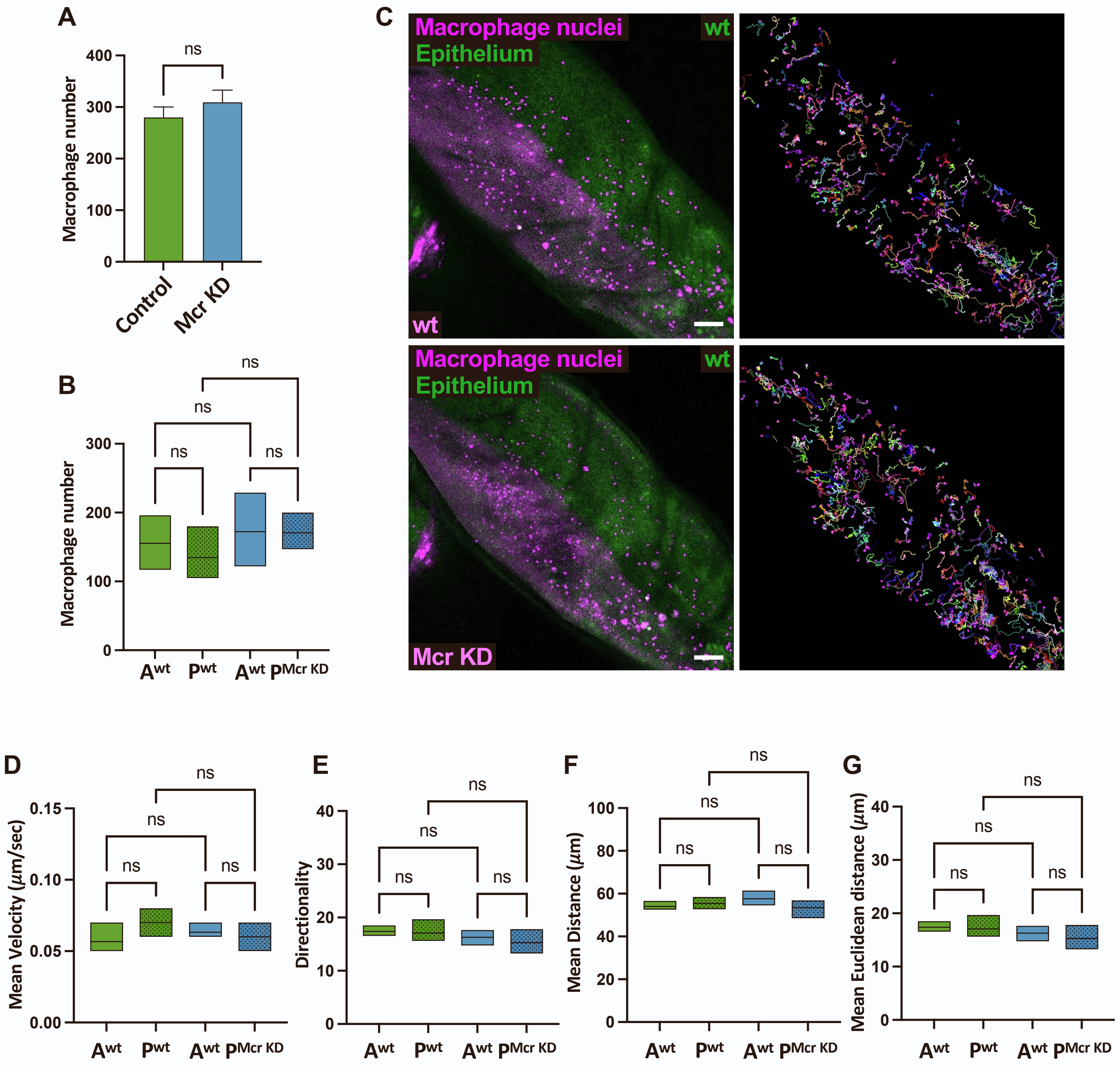

A
Macrophage number
ns
Control
Mcr KD
B
Macrophage number
ns
A^wt
P^wt
A^wt P^Mcr KD
C
Macrophage nuclei
Epithelium
wt
Mcr KD
D
Mean Velocity (μm/sec)
E
Directionality
F
Mean Distance (μm)
G
Mean Euclidean distance (μm)

Figure S1: Mcr KD in the posterior compartment of the pupal wing does not affect homeostatic macrophage behaviour. Related to Figure 1.

(A-B) Quantification of number of macrophages (A) and their distribution along the anterior and posterior compartments (B) in pupal wings 18h APF in control and upon Mcr KD in the posterior compartment, in the absence of a wound. **(C)** Representative frame of a time-lapse movie of macrophage nuclei (magenta) in an unwounded pupal wing 18h APF, and relative tracks (right panels) in control and upon Mcr KD in the posterior compartment. Scale bar 50 μm. green: epithelium **(D-G)** Quantification of macrophage mean velocity (D), Directionality (E), Mean distance (F) and Mean Euclidean distance (G) across the Anterior (A) and Posterior (P) compartments as in A.

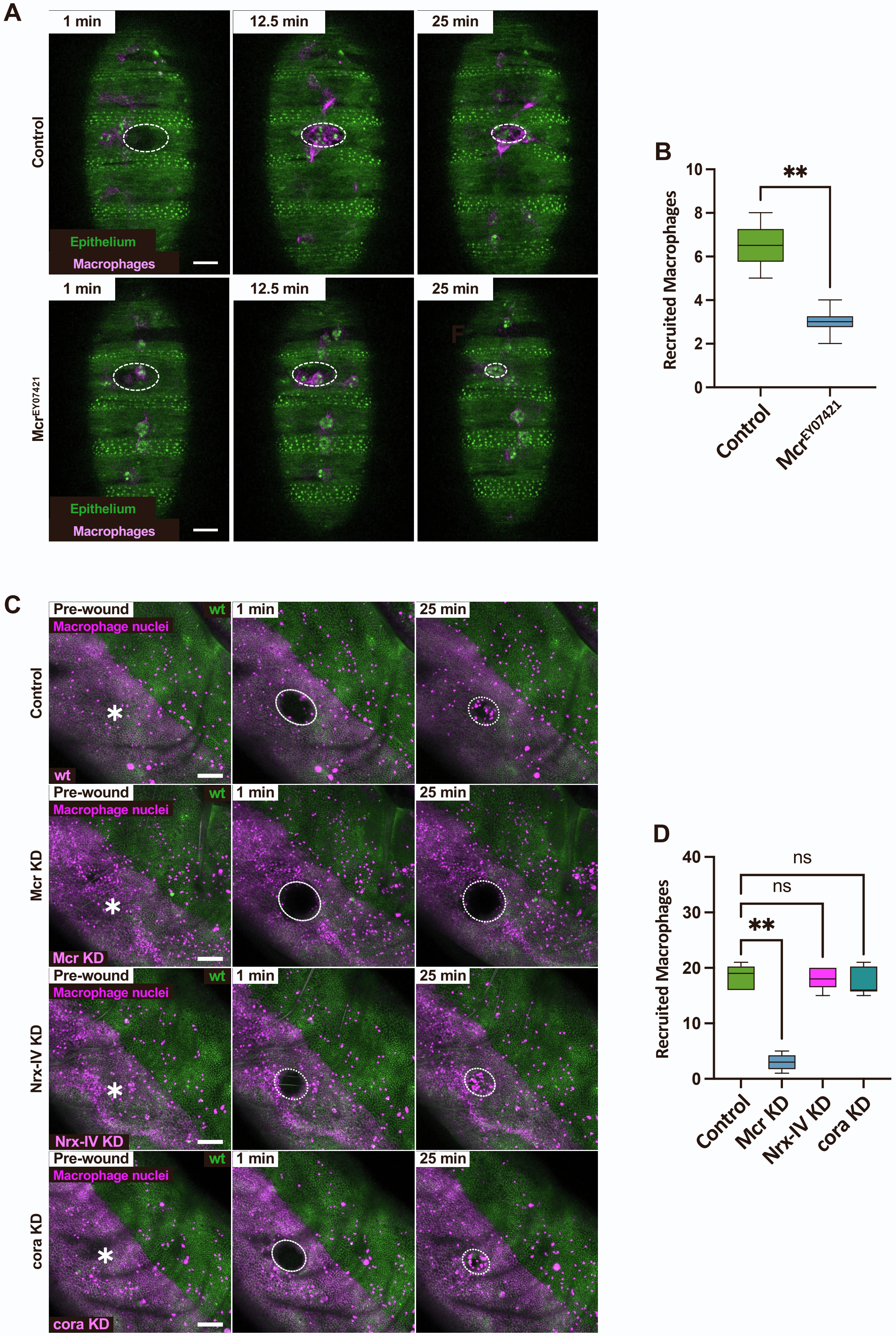

A
1 min
12.5 min
25 min
Control
Epithelium
Macrophages
Mcr^EY07421
B
Recruited Macrophages
**
Control
Mcr^EY07421
C
Pre-wound
wt
Macrophage nuclei
1 min
25 min
Control
Mcr KD
Nrx-IV KD
cora KD
D
ns
ns
**
Recruited Macrophages
Control
Mcr KD
Nrx-IV KD
cora KD

Figure S2: Epithelial Mcr is required for efficient macrophage recruitment to wounds independent of function in septate junctions. Related to Figure 1.

(A-B) Confocal time-lapse microscopy of wounded stage 15 control embryos and $Mcr^{EY07421}$ mutants (A) and quantification of macrophage number recruited to the wound site (B). **(C-D)** Confocal time-lapse microscopy of pupal wing 18h APF, showing macrophage nuclei (dark magenta dots) recruited to the wound site upon knockdown of the septate junction components Mcr, Nrx-IV and cora (C) and quantification of macrophage number recruited to the wound site (D).

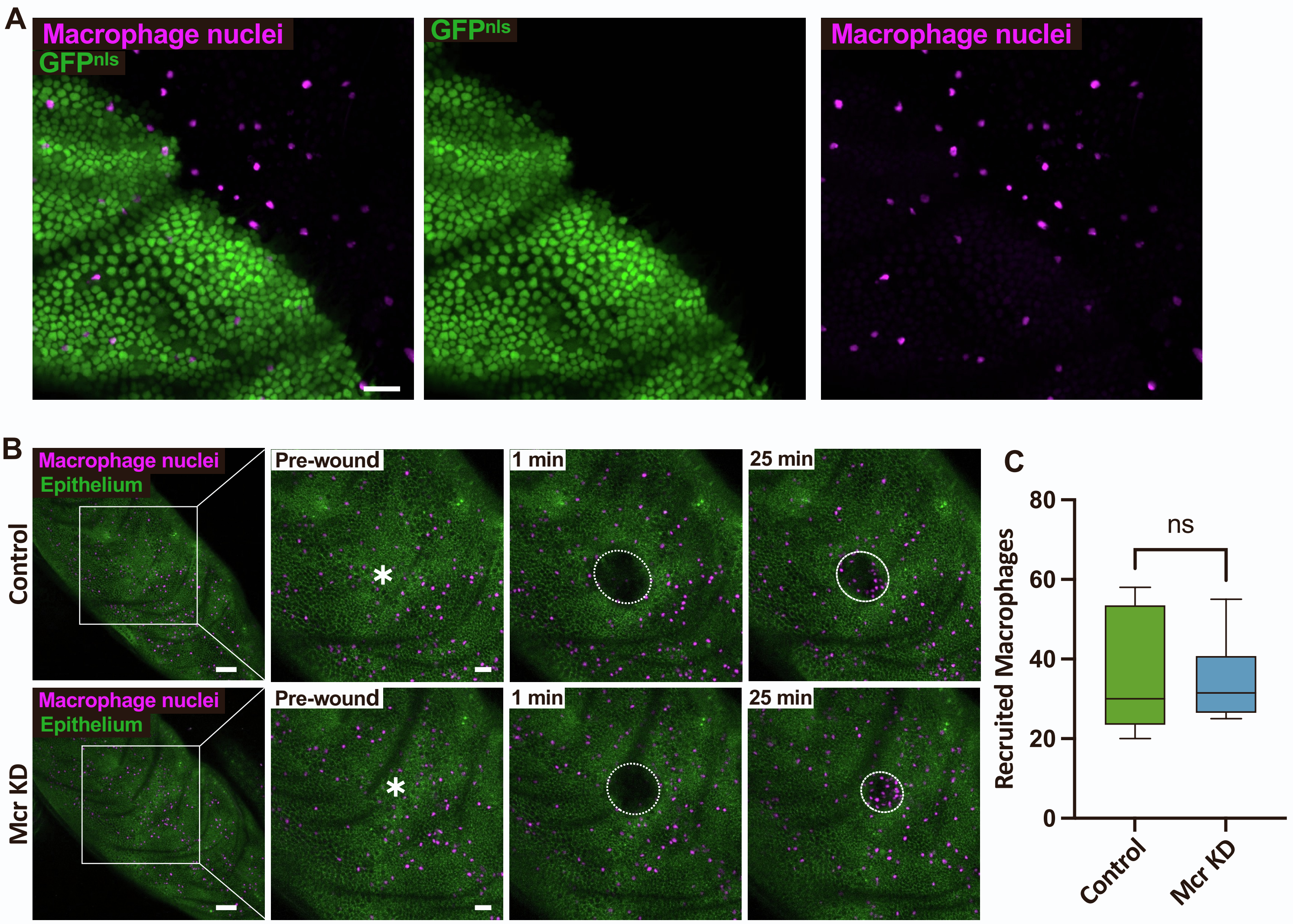
A
Macrophage nuclei
GFP^{nls}
GFP^{nls}
Macrophage nuclei
B
Control
Mcr KD
Macrophage nuclei
Epithelium
Pre-wound
1 min
25 min
C
Recruited Macrophages
0
20
40
60
80
ns
Control
Mcr KD

Figure S3: Mcr KD within macrophages has no effect on wound recruitment. Related to Figure 1.

(A) Representative confocal projection showing the engrailed driver expressing a nuclear GFP (green) construct that has no overlap with the macrophage population (magenta). Scale bar 20 μm. **(B)** Time lapse imaging of macrophage recruitment to the wound at 1 and 25 min after wounding showing no defects in macrophage recruitment after macrophage specific Mcr KD. Scale bars: 50 μm (left panels), 20 μm (right panels); asterisks: wound location; green: epithelium. **(C)** Quantification of the macrophages recruited to the wound as in B.

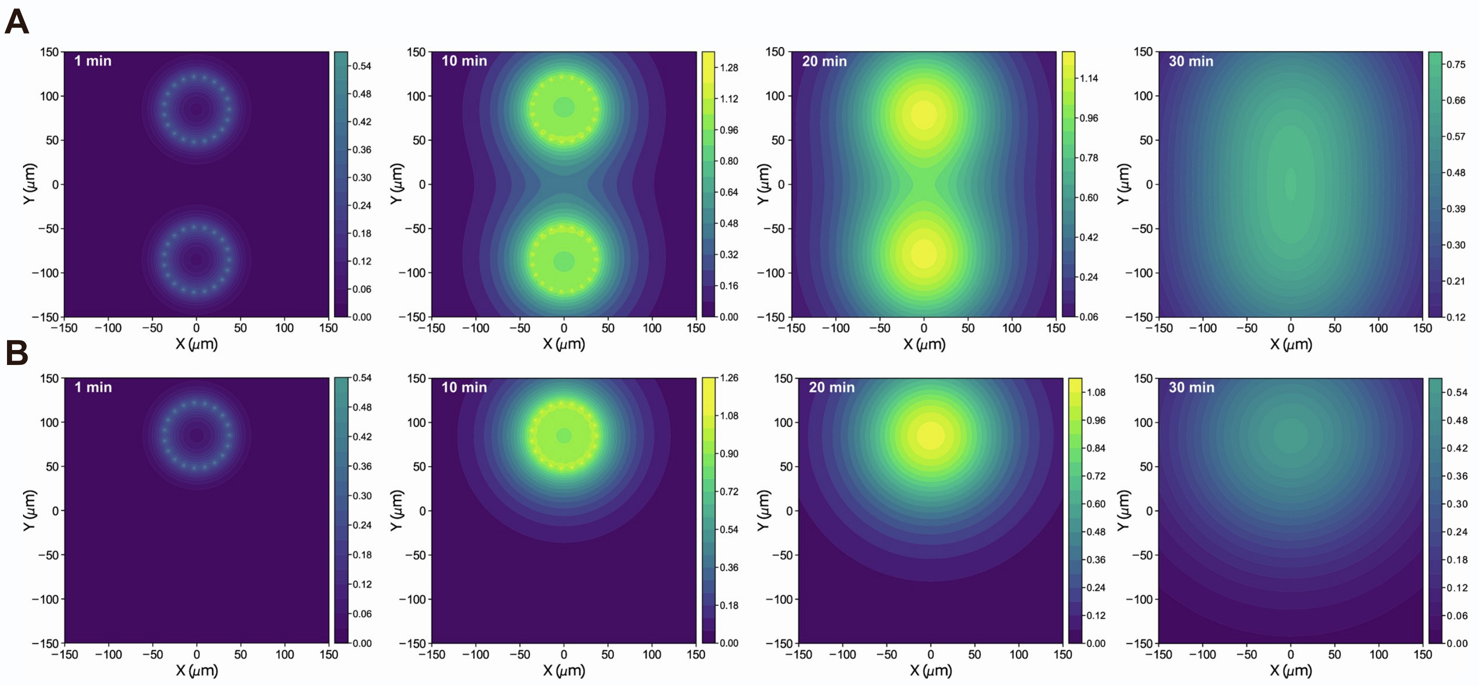
A
B
1 min
10 min
20 min
30 min
X (μm)
Y (μm)

Figure S4: Predicted chemoattractant distribution between two competing wounds. Related to Figure 4.

(A) Snapshots of predicted chemoattractant distribution (in arbitrary units) around and between two wounds at 1, 10, 20, and 30 min post wounding, using the same parameters as in Figure 4, and approximating the wounds as circles of point sources along the wound edge. **(B)** Snapshots of predicted chemoattractant distribution and gradient thereof, as in A but now with one Control wound and one lacking chemoattractant.

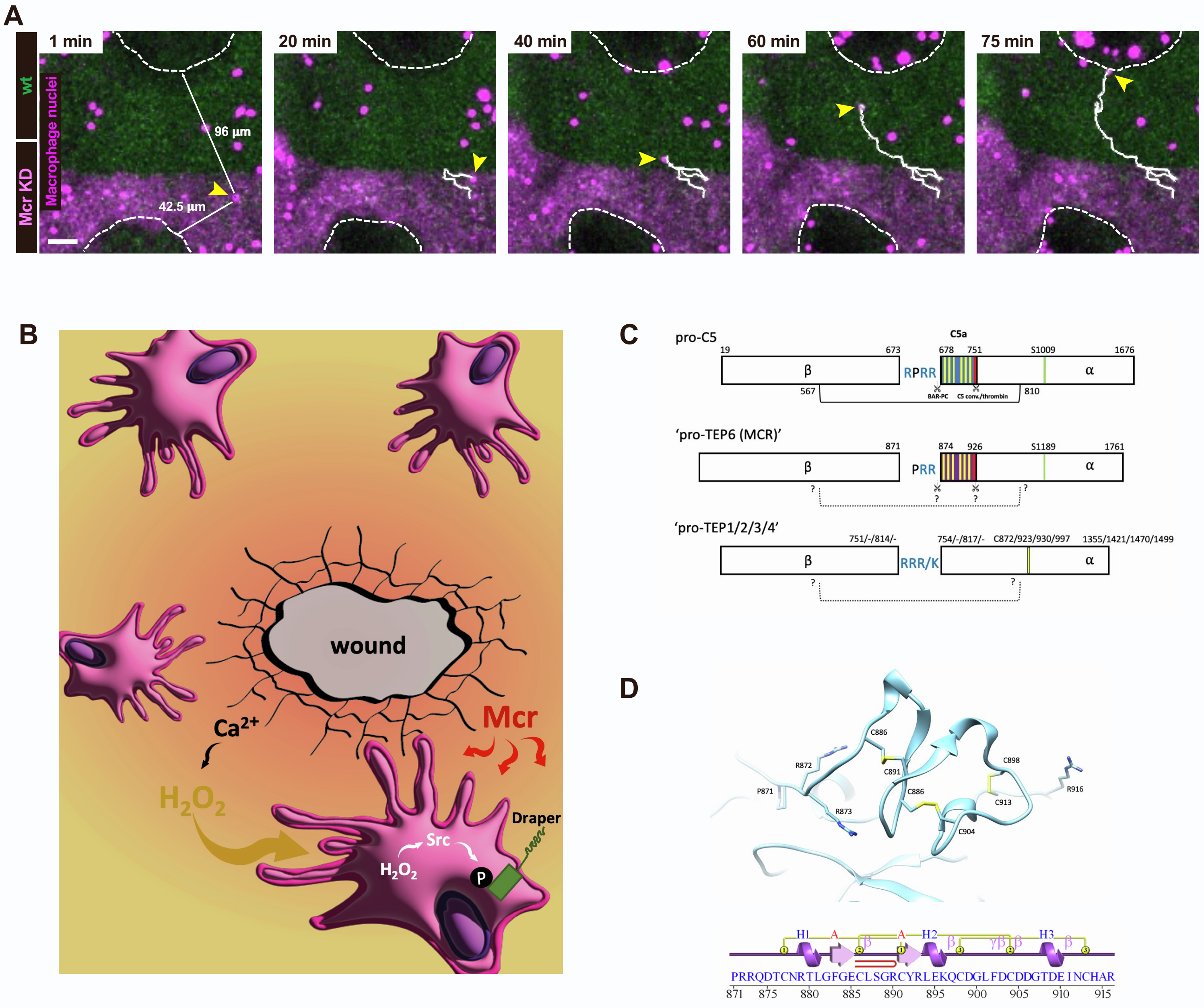

A
1 min
20 min
40 min
60 min
75 min
wt
Mcr KD
Macrophage nuclei
96 µm
42.5 µm
B
wound
Ca2+
H2O2
Mcr
Src
H2O2
P
Draper
C
pro-C5
C5a
19
673
678
751
S1009
1676
β
RPRR
α
567
BAR-PC
C5 conv./thrombin
810
'pro-TEP6 (MCR)'
871
874
926
S1189
1761
β
PRR
α
'pro-TEP1/2/3/4'
751/-/814/-
754/-/817/-
C872/923/930/997
1355/1421/1470/1499
β
RRR/K
α
D
C886
C891
C886
C898
R872
P871
R873
C913
R916
C904
H1
A
A
H2
H3
PRRQDTCNRTLGFGECLSGRCYRLEKQCDGLFDCDDGTDEINCHAR
871 875 880 885 890 895 900 905 910 915

Figure S5: Mcr operates as a macrophage chemoattractant *in vivo.* Related to Figure 4.

(A) Timelapse and overlap tracking (white line) of a single macrophage (yellow arrowhead) in close proximity to a wound generated in a Mcr-deficient epithelium that is attracted by a more distant wild-type wound. Scale bar 20 μm. **(B)** Schematic representation of the proposed role of Mcr (red) in coordinating with H_2O_2 to drive macrophage recruitment to wounds **(C)** Sequence comparison of human C5 with *Drosophila* TEPs 1-6 highlighting the presence of a C5a-like cysteine-rich region in Mcr/TEP6. **(D)** Alphafold prediction and topology diagram of the C5a-like sequence region in Mcr/TEP6. Shown are the positions of the potential basic amino acid residue-specific proprotein convertase (BAR-PC) cleavage sequence (PRR, upper panel) and serine protease cleavage site (R916, lower panel). The predicted structure of the MCR/TEP6 C5a-like region resembles the structure of cysteine-rich complement-type repeats seen in lipoprotein-related receptor proteins (DALI analysis), stapled together by three disulfide bonds. The ribbon diagram was created using UCSF Chimera and the topology image was generated using PDBsum (EMBL-EBI).

List of Genotypes		
1B-D	Control	; ubi-DE-cadherin-GFP, En-Gal4, uas-Moe-Cherry/+; Srp-3XmCherry/+
	Mcr KD	; ubi-DE-cadherin-GFP, En-Gal4, uas-Moe-Cherry/+; Srp-3XmCherry/ uas-Mcr RNAi
1E-M	Control	; ubi-DE-cadherin-GFP, En-Gal4, uas-Moe-Cherry/+; Srp-H2A-cherry/+
	Mcr KD	; ubi-DE-cadherin-GFP, En-Gal4, uas-Moe-Cherry/+; Srp-H2A-cherry/ uas-Mcr RNAi
2B-E	Control	; ubi-DE-cadherin-GFP, En-Gal4, uas-Moe-Cherry/+; Srp-H2A-cherry/+
	Mcr KD	; ubi-DE-cadherin-GFP, En-Gal4, uas-Moe-Cherry/+; Srp-H2A-cherry/ uas-Mcr RNAi
3C-H	Control	; ubi-DE-cadherin-GFP, En-Gal4, uas-Moe-Cherry/+; Srp-H2A-cherry/+
	Mcr KD	; ubi-DE-cadherin-GFP, En-Gal4, uas-Moe-Cherry/+; Srp-H2A-cherry/ uas-Mcr RNAi
4C-G	Control	; ubi-DE-cadherin-GFP, En-Gal4, uas-Moe-Cherry/+; Srp-H2A-cherry/+
	Mcr KD	; ubi-DE-cadherin-GFP, En-Gal4, uas-Moe-Cherry/+; Srp-H2A-cherry/ uas-Mcr RNAi
4H, I	Control	; EMS-Gal4, uas-GMA/+; Srp-H2A-cherry/+
	Mcr OE	; EMS-Gal4, uas-GMA/uas-Mcr; Srp-H2A-cherry/+
S1A-G	Control	; ubi-DE-cadherin-GFP, En-Gal4, uas-Moe-Cherry/+; Srp-H2A-cherry/+
	Mcr KD	; ubi-DE-cadherin-GFP, En-Gal4, uas-Moe-Cherry/+; Srp-H2A-cherry/ uas-Mcr RNAi
S2A, B	Control	w; e22c-Gal4, uas-Moe-Cherry, Srp-GMA; Srp-GMA
	$Mcr^{EY07421}$	w; e22c-Gal4, uas-Moe-Cherry, Srp-GMA, $Mcr^{EY07421}$; Srp-GMA
S2C, D	Control	; ubi-DE-cadherin-GFP, En-Gal4, uas-Moe-Cherry/+; Srp-H2A-cherry/+
	Mcr KD	; ubi-DE-cadherin-GFP, En-Gal4, uas-Moe-Cherry/+; Srp-H2A-cherry/ uas-Mcr RNAi
	Nrx-IV KD	; ubi-DE-cadherin-GFP, En-Gal4, uas-Moe-Cherry/Nrx-IV RNAi; Srp-H2A-cherry/ +
	cora KD	; ubi-DE-cadherin-GFP, En-Gal4, uas-Moe-Cherry/cora RNAi; Srp-H2A-cherry/ +
S3A		; En-Gal4, uas-GFP^{nls}/+; Srp-H2A-cherry/+
S3B, C	Control	Nrg^{GFP}; Srp-Gal4.2/+; Srp-H2A-cherry/+
	Mcr KD	Nrg^{GFP}; Srp-Gal4.2/+; Srp-H2A-cherry/uas-Mcr RNAi
S5A		; ubi-DE-cadherin-GFP, En-Gal4, uas-Moe-Cherry/+; Srp-H2A-cherry/ uas-Mcr RNAi

Table S1 - List of Genotypes. Related to figure 1-4 and S1-5.

Experimental details				
Figure	Genotype	Sample	Macrophage number/tracks	ROI size
1B-D	Control	1	14	354μmx354μm
		2	22	
		3	15	
		4	18	
		5	16	
	Mcr KD	1	2	
		2	2	
		3	2	
		4	3	
		5	1	
1E-M	Control	1	62	150μmx150μm
		2	38	
		3	41	
		4	58	
		5	44	
	Mcr KD	1	62	
		2	67	
		3	42	
		4	51	
2B-E	Control	1	302	
		2	294	
		3	398	
		4	270	
		5	269	
		6	330	
	Mcr KD	1	432	
		2	315	
		3	318	
		4	317	
		5	329	
3C, D	Control	1	204	300μmx300μm
		2	181	
		3	184	
		4	159	
		5	242	
		6	202	
	Mcr KD	1	136	
		2	167	
		3	197	
		4	168	
		5	138	
		6	116	
3E-H	Control	1	A: 122 P: 90	300μmx300μm
		2	A: 87 P: 80	
		3	A: 118 P: 121	
		4	A: 103 P: 146	

	Mcr KD	1	A: 69 P: 79	
		2	A: 109 P: 78	
		3	A: 71 P: 92	
		4	A: 47 P: 76	
4C-E	Control	1	162	200μmx170μm
		2	146	
		3	123	
		4	178	
	Mcr KD	1	97	
		2	124	
		3	106	
		4	194	
4F, G	Control	1	24	147μmx85μm
		2	22	
		3	23	
		4	30	
	Mcr KD	1	25	
		2	20	
		3	34	
		4	34	
4H, I	Control	1	20	120μmx80μm
		2	22	
		3	23	
		4	21	
		5	20	
		6	21	
	Mcr OE	1	38	
		2	38	
		3	36	
		4	36	
		5	36	
		6	37	
S1A-G	Control	1	A: 196 P: 180	
		2	A: 153 P: 119	
		3	A: 117 P: 105	
	Mcr KD	1	A: 122 P: 147	
		2	A: 166 P: 165	
		3	A: 229 P: 200	
S2A, B	Control	1	6	
		2	8	
		3	7	
		4	6	
		5	7	
		6	5	
	$Mcr^{EY07421}$	1	2	
		2	3	
		3	3	

		4	4	
		5	3	
		6	3	
S2C, D	Control	1	20	
		2	16	
		3	20	
		4	16	
		5	21	
		6	18	
	Mcr KD	1	3	
		2	1	
		3	4	
		4	3	
		5	5	
		6	2	
	Nrx IR	1	20	
		2	20	
		3	17	
		4	15	
		5	17	
		6	19	
	cora IR	1	15	
		2	15	
		3	16	
		4	21	
		5	20	
		6	16	
S3B, C	Control	1	30	
		2	20	
		3	49	
		4	58	
		5	27	
	Mcr KD	1	25	
		2	36	
		3	55	
		4	34	
		5	29	
		6	27	

A: Anterior P: Posterior

Table S2 - Experimental details. Related to Figure 1-4 and S1-5.
