# Supplementary material for: Drosophila complement-like Mcr acts as a wound-induced inflammatory chemoattractant
Source: Curr Biol. 2025 Mar 18;35(7):1656–1664.e4. doi: 10.1016/j.cub.2025.02.036 (PMC12254109; doi:10.1016/j.cub.2025.02.036)
Supplement: Document S2. Article plus supplemental information [file mmc7.pdf]

Current Biology

Report

Drosophila complement-like Mcr acts as a wound-induced inflammatory chemoattractant

Graphical abstract

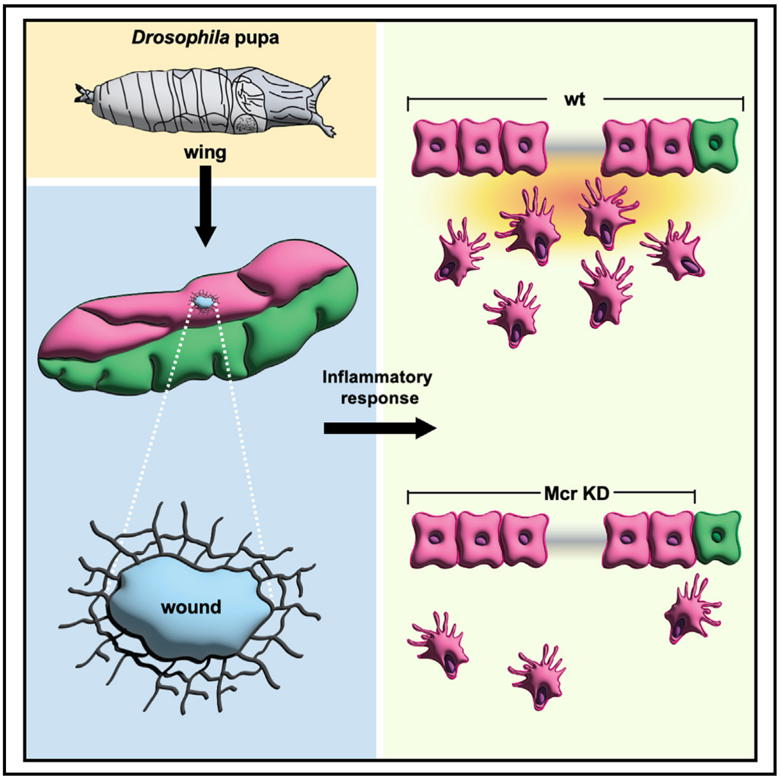

Authors

Luigi Zechini, Henry Todd,
Thibaut Sanchez, ...,
Linus J. Schumacher,
Alessandro Scopelliti, Will Wood

Correspondence

linus.schumacher@ed.ac.uk (L.J.S.),
alessandro.scopelliti@ed.ac.uk (A.S.),
w.wood@ed.ac.uk (W.W.)

In brief

Zechini et al. identify macroglobulin complement-related (Mcr) as a wound-induced chemotactic signal essential for macrophage recruitment to sites of sterile damage in *Drosophila*. Combining genetics, live imaging, and mathematical modeling, they investigate the role of Mcr in ensuring a rapid and robust inflammatory response *in vivo*.

Highlights

- Epithelial Mcr is essential for macrophage migration to wounds in *Drosophila*
- Mcr operates as an immediate damage-induced macrophage chemoattractant
- Mcr's inflammatory role parallels the function of mammalian complement C5a

Zechini et al., 2025, Current Biology *35*, 1656–1664
April 7, 2025 © 2025 The Author(s). Published by Elsevier Inc.
https://doi.org/10.1016/j.cub.2025.02.036

CellPress

CellPress
OPEN ACCESS

Current Biology

Report

Drosophila complement-like Mcr acts as a wound-induced inflammatory chemoattractant

Luigi Zechini,[1] Henry Todd,[1] Thibaut Sanchez,[1] Daniel R. Tudor,[1] Jennie S. Campbell,[1] Edward Antonian,[1] Stephen J. Jenkins,[1] Christopher D. Lucas,[1] Andrew J. Davidson,[2] Jean van den Elsen,[3] Linus J. Schumacher,[1,4,*] Alessandro Scopelliti,[1,*] and Will Wood[1,5,*]

[1]Institute for Regeneration and Repair, University of Edinburgh, Edinburgh BioQuarter, 4-5 Little France Drive, Edinburgh EH16 4UU, UK
[2]Wolfson Wohl Cancer Research Centre, School of Cancer Sciences, University of Glasgow, Garscube Estate, Switchback Road, Bearsden, Glasgow G61 1BD, UK
[3]Department of Life Sciences, University of Bath, Claverton Down, Bath BA2 7AY, UK
[4]School of Mathematics and Maxwell Institute for Mathematical Sciences, University of Edinburgh, Edinburgh EH9 3FD, UK
[5]Lead contact
*Correspondence: linus.schumacher@ed.ac.uk (L.J.S.), alessandro.scopelliti@ed.ac.uk (A.S.), w.wood@ed.ac.uk (W.W.)
https://doi.org/10.1016/j.cub.2025.02.036

SUMMARY

Sterile tissue injury is accompanied by an acute inflammatory response whereby innate immune cells rapidly migrate to the site of injury guided by pro-inflammatory chemotactic damage signals released at the wound. Understanding this immune response is key to improving human health, and recent advances in imaging technology have allowed researchers using different model organisms to observe this inflammatory response *in vivo*. Over recent decades, offering a unique combination of live time-lapse microscopy and genetics, the fruit fly *Drosophila* has emerged as a powerful model system to study inflammatory cell migration within a living animal.[1–4] However, we still know relatively little regarding the identity of the earliest signals that drive this immune cell recruitment and the mechanisms by which they act within the complex, *in vivo* setting of a multicellular organism. Here, we couple the powerful genetics and live imaging of *Drosophila* with mathematical modeling to identify the fly complement ortholog—macroglobulin complement-related (Mcr)—as an early, wound-induced chemotactic signal responsible for the inflammatory recruitment of immune cells to injury sites *in vivo*. We show that epithelial-specific knockdown of Mcr suppresses the recruitment of macrophages to wounds and combine predictive mathematical modeling with *in vivo* genetics to understand macrophage migration dynamics following manipulation of this chemoattractant. We propose a model whereby Mcr operates alongside hydrogen peroxide to ensure a rapid and efficient immune response to damage, uncovering a novel function for this protein that parallels the chemotactic role of the complement component C5a in mammals.

RESULTS AND DISCUSSION

Laser-induced wounding in the fly triggers an immediate and efficient chemotactic response from inflammatory immune cells that rapidly migrate into the site of aseptic tissue damage in a process mimicking mammalian inflammation.[5] This chemotactic response has been shown to be dependent on the production of hydrogen peroxide (H_2O_2) at the wound, and for several years, it was thought to be the chemotactic factor guiding macrophages to the wound site.[2,6,7] However, subsequent work using mathematical modeling showed this not to be the case.[8] It now appears that H_2O_2 operates as a "priming" signal involving the phosphorylation of the receptor Draper,[9] readying the macrophages for response to a second, as yet unknown, damage-induced chemotactic signal. One prime candidate for this role is the *Drosophila* complement ortholog—macroglobulin complement-related (Mcr)—not least because the complement system is a long-established regulator of immune cell biology but, critically, also because *Drosophila* Mcr has previously been shown to interact with and act upstream of Draper activation *in vivo*.[10]

To begin investigating a chemotactic role for Mcr following wounding in the fly, we used the pupal wing, where laser wounds trigger a robust inflammatory recruitment of macrophages to the site of damage within minutes.[8] We used an RNAi construct targeting Mcr (upstream activating sequence [UAS]-Mcr RNAi), driven by the engrailed-Gal4 construct, to knock down Mcr expression in the posterior compartment of the *Drosophila* pupal wing epithelium (Figure 1A). We found that wounding in the Mcr knockdown tissue led to a significant reduction in the number of macrophages recruited to the wound site within 20 min when compared with controls (Figures 1B–1D; Video S1), whereas macrophage number, distribution along the antero-posterior compartments, and migratory behavior in unwounded wings was entirely normal (Figures S1A–S1G). Importantly, we observed an analogous reduction in the number of macrophages recruited to epidermal wounds made to embryos mutant for Mcr (*Mcr*[EY07421]) when compared with wild-type

 © 2025 The Author(s). Published by Elsevier Inc.
This is an open access article under the CC BY license (http://creativecommons.org/licenses/by/4.0/).

CellPress
OPEN ACCESS

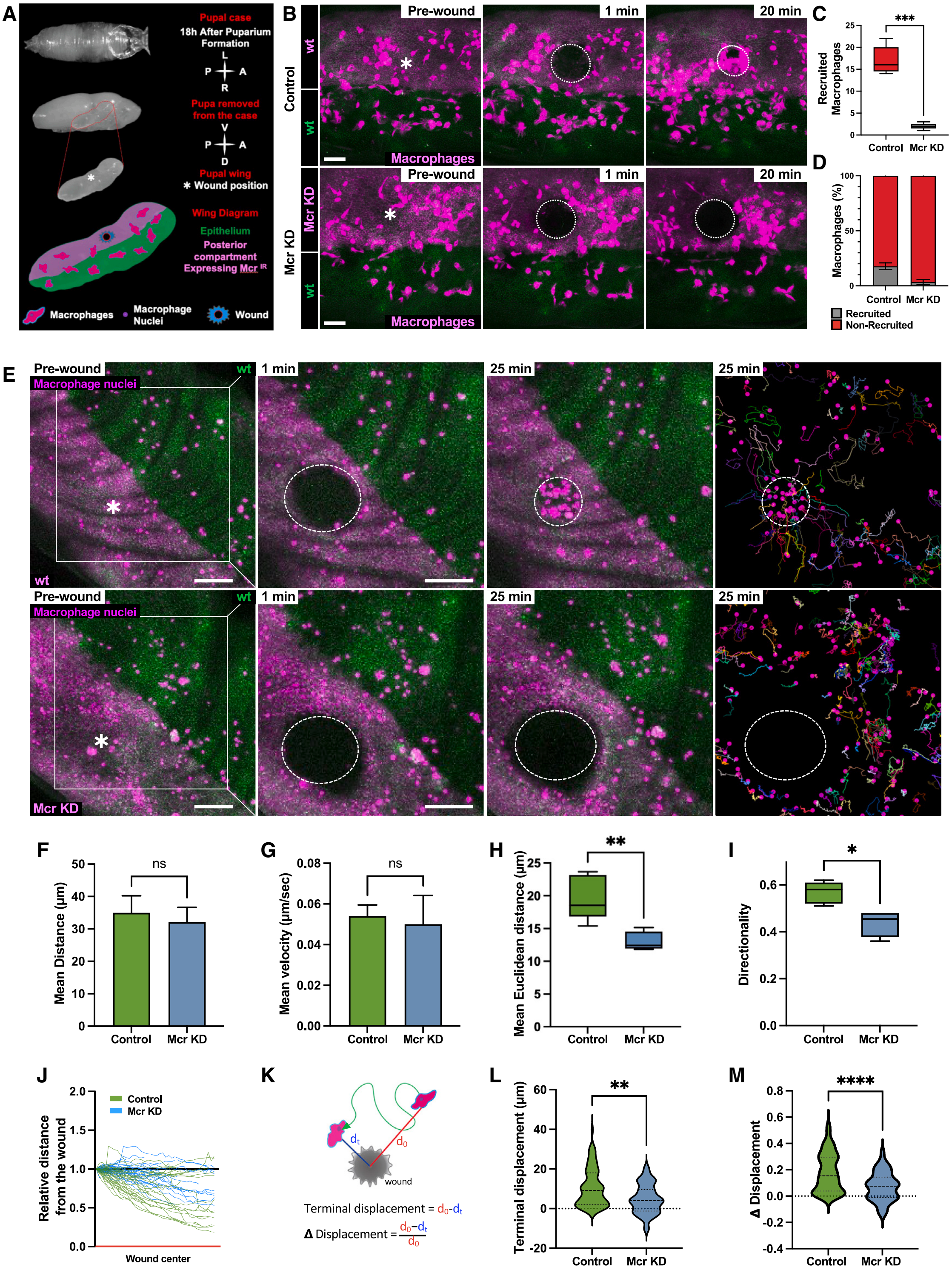

Figure 1. Epithelial knockdown of Mcr suppresses macrophage recruitment to wounds

(A) Schematic representation of pupae 18 h after puparium formation (APF). From top to bottom: pupal case, top view. Pupa dissected from the case, lateral view. Pupal wing, lateral view. Asterisk highlights the anatomical region where the wound is performed. Wing schematic, showing the antero-posterior compartments and wound position.

(B) Confocal time-lapse microscopy of pupal wing 18 h APF, showing macrophage (magenta) recruitment upon wounding in control conditions (upper) and after knockdown (KD) of Mcr (lower) in the posterior compartment (light magenta). For all microscope images, time points refer to minutes post wounding, asterisks indicate wound site, and dashed lines show wound outline.

(legend continued on next page)

control embryos (Figures S2A and S2B). Because Mcr is a known transmembrane septate-junction-associated protein whose absence leads to barrier defects,[11] we wanted to investigate whether the defective macrophage recruitment to wounds was a consequence of disrupted epithelial barrier function. To this end, we made wounds to pupal wings following independent RNAi knockdown of two other essential septate junction components (Nrx-IV and cora) using validated reagents[12,13] and monitored the resulting macrophage migration. We observed no effect on macrophage recruitment to wounds following knockdown of either of these components (Figures S2C and S2D; Video S2), demonstrating that altering the structure and function of the epithelium is not responsible for the macrophage recruitment phenotype we observed following Mcr knockdown and that Mcr's function in driving macrophage recruitment to wounds is completely independent of its role as a septate junction component.

To gain further insights into the observed macrophage recruitment defect following Mcr knockdown, we took advantage of constitutive nuclear-labeled immune cells, allowing us to track with high fidelity their migratory behavior using time-lapse microscopy (Figure 1E; Video S3). Our live imaging analysis in wounded pupal wings revealed that, despite migrating at a comparable speed and traveling comparable distances (Figures 1F and 1G), macrophage behavior after wounding Mcr-depleted epithelia is more erratic (Figure 1H) and less directional (Figure 1I). Further analysis showed that, in the absence of epithelial Mcr, macrophages fail to efficiently detect the site of damage (Figure 1J), with fewer macrophages able to re-orientate and migrate toward the wound site (Figures 1J–1M). Because Mcr has also previously been reported to be expressed by *Drosophila* macrophage cell lines,[14,15] we both verified that our epithelial-specific expression system exhibits no off-target expression in the macrophage lineage (Figure S3A) and demonstrated that macrophage recruitment to wounds was unaffected when Mcr was knocked down in macrophages (Figures S3B and S3C). Taken together, these results confirm that the wound recruitment phenotype observed was due to a role of Mcr within the epithelium rather than within the migrating macrophages themselves.

To directly test whether the macrophage migration phenotype we observed is consistent with a chemotactic role for Mcr following wounding, we turned to mathematical modeling. In previous studies, mathematical modeling has proven invaluable in delivering detailed information about the wound attractant signal by applying a two-step computational inference pipeline[8] (Figure 2A). First, bias toward the wound location and persistence of movement direction between timesteps is calculated from cell-tracking data to infer the parameters of a biased-persistent random walk model (Figures 2A and 2B). This is done for a range of space and time bins, and from the spatiotemporal variation of the bias, we infer the diffusivity of a chemoattractant signal using a second mathematical model of signal production and diffusion. We reproduced and refined previous modeling approaches as open-source pipeline implemented in the Python programming language (STAR Methods). Consistent with previous modeling approaches,[8] we found that the wound-induced damage signal diffuses at a speed of 100–300 μm^2/min (Figures 2D and 2E) and leads to a rapid response from macrophages up to 100 μm from the wound over the first 20 min post wounding (Figure 2C). The same analysis of macrophage migration patterns in the presence of wounds made to Mcr knockdown epithelium detected no evidence for a signal with well-defined diffusivity, consistent with Mcr being the immediate chemoattractant signal released from wounds (Figures 2B–2D).

We then used our mathematical model to predict how immune cells would behave in more complex scenarios, where the source of wound chemoattractant is manipulated or where cells are exposed to competing wound attractants. Previous work has shown that the wound attractant cue is released from wound margin cells rather than the wounded tissue per se.[8] Based on this, we used modeling to predict the response of macrophages to a wound where only half the wound margin is producing the attractant signal whereas the other half does not. When this scenario is modeled *in silico*, we found that there is a stronger and steeper gradient on the side of the wound producing the chemoattractant, whereas a shallower gradient extends on the other side of the wound (Figures 3A and 3B). In order to test whether we observe a corresponding migratory pattern in macrophages *in vivo* in such a scenario, we generated laser wounds across the antero-posterior wing boundary, such that the posterior half of the wound edge epithelium lacks Mcr while the anterior half is wild type. Time-lapse imaging of the resulting wounds revealed that the responding macrophages behaved exactly as predicted by the model, with cells migrating rapidly to the wild-type half of the wound while largely ignoring the Mcr knockdown wound margin (Figures 3C and 3D; Video S4). This defect leads to a clear imbalance of macrophage recruitment during the initial 20 min following wounding (Figures 3C and 3D; Video S4). Quantitative analysis also shows that macrophages migrating to the wild-type half of the wound do so in a highly directional manner, being able to re-orientate and migrate toward the wound site as opposed to those close to the Mcr knockdown half of the wound that show reduced directionality despite moving at the same speed (Figures 3E–3H).

(C) Quantification of the number of macrophages recruited at the wound site after 20 min as in (B).
(D) Ratio of recruited to non-recruited macrophages following wounding as in (B).
(E) Confocal time-lapse microscopy of pupal wing 18 h APF, showing macrophage nuclei (dark magenta dot) recruited to the wound site and tracking of macrophages for 25 min after wounding (right). Scale bars, 50 μm.
(F–I) Behavior of tracked macrophages within 150 μm of the wound center: (F) mean distance, (G) mean velocity, (H) mean Euclidean distance, and (I) directionality.
(J) Representative individual macrophage tracks relative to the center of the wound.
(K) Graphical representation defining the parameters for (H) and (I).
(L) Terminal displacement.
(M) Δ Displacement of tracked macrophages. Data are represented as mean ± SEM. Dotted lines in (L) and (M) represent the median. Asterisks indicate significant differences (*$p < 0.05$; **$p < 0.01$; ***$p < 0.001$; ****$p < 0.0001$; ns, not significant).
See also Figures S1–S3; Videos S1, S2, and S3; and Tables S1 and S2.

CellPress
OPEN ACCESS

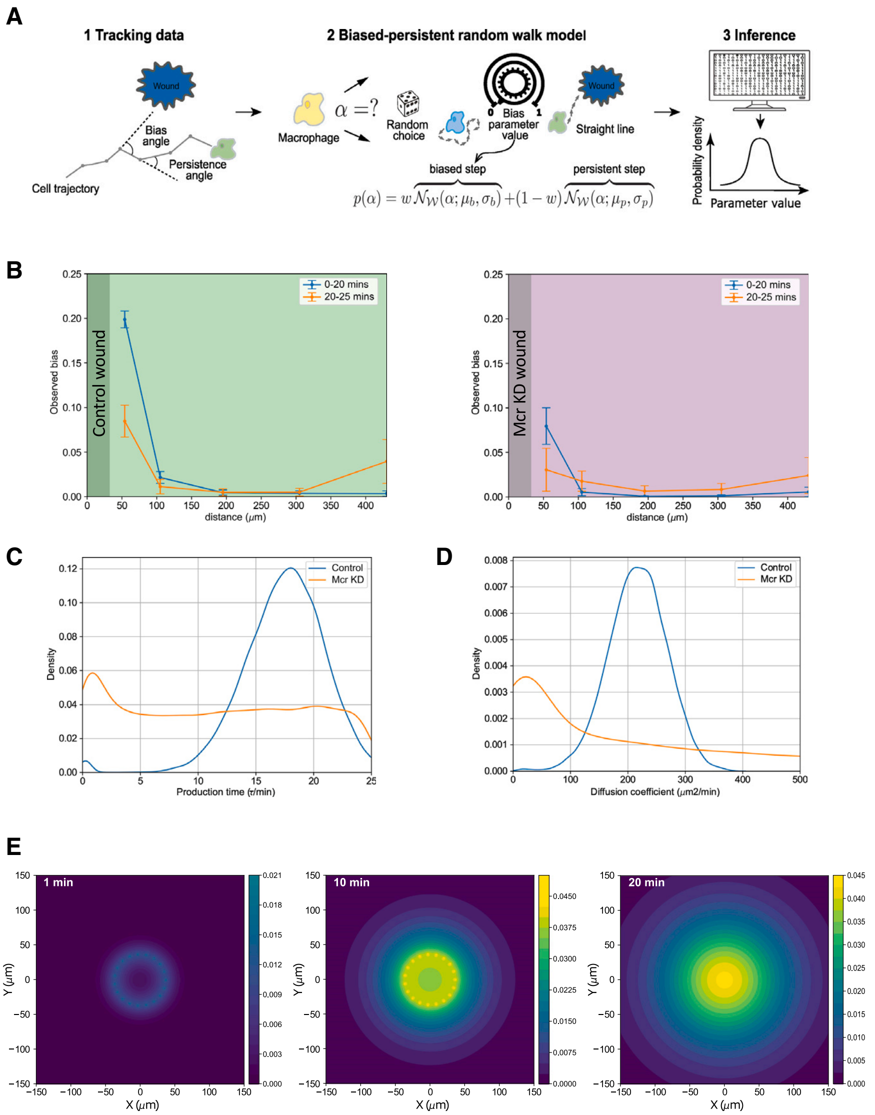

Figure 2. Mathematical models and statistical inference of macrophage migration and chemoattractant production-diffusion

(A) Schematic of workflow to infer cell migration parameters. Bias and persistence angles are calculated from cell-tracking data (1) and fed into biased-persistent random walk model (2) (STAR Methods). The bias parameter b sets the width of a normal distribution for the bias angle through $\sigma_b^2 = -2\log(b)$, and similarly for the persistence parameter p, whereas the weight parameter w sets the balance between biased and persistent steps. A posterior probability distribution over the parameters b, p, w is calculated through Bayesian inference (3).

(B) Observed bias, $w \times b$, inferred from cell-tracking data vs. distance from wound center at different times after wounding in control (left) and Mcr KD (right) samples. Error bars show one standard deviation around the mean of the posterior distribution for each bin. Background color indicates the control (green) and Mcr KD (magenta) epithelium and wound area (gray).

(C) Posterior distribution of chemoattractant production time, τ, inferred from the spatiotemporal variation of the observed bias, assuming secretion from the wound at a constant rate from $t = 0$ until $t = \tau$ (STAR Methods). The inferred distribution for the control samples is in agreement with previously published data,[8] whereas the result for Mcr KD samples shows no evidence for chemoattractant production within the duration of the videos analyzed (25 min).

(D) Posterior distribution of chemoattractant diffusivity, D, inferred from the spatiotemporal variation of the observed bias. The inferred distribution for the control samples is in agreement with previous published data,[8] whereas the result for Mcr KD samples does not deviate from the uniform prior distribution, indicating no evidence for any chemoattractant signal in Mcr KD conditions.

(E) Snapshots of predicted chemoattractant distribution (in arbitrary units) at 1, 10, and 20 min post wounding, using the mode of the posterior distribution as parameters and approximating the wound as a circle of point sources along the wound edge.

See also Tables S1 and S2.

We next used our modeling to predict what would happen if macrophages were faced with two competing wounds made simultaneously. Consistent with previous work,[8] our model predicts that wounds made at the same time lead to overlapping chemotactic gradients in the inter-wound region that compete with one another for macrophage attention. This leads to a reduction in macrophage migration bias in this region, where macrophages remain "confused" and fail to migrate to either wound (Figures 4A and S4A). Additionally, we model the case of only one of the two wounds being able to generate the chemotactic signal. In such a scenario, the model predicts the generation of a steep chemotactic gradient only on the side of the wound producing the chemoattractant, leading the macrophages in the inter-wound region to respond exclusively to the attractant-producing wound and ignore the wound unable to release chemoattractant cues (Figures 4B and S4B). We then validated these predictions experimentally *in vivo* by generating two simultaneous wounds of equal size in wild-type tissue and imaging the responding macrophages. As predicted, tracking the resulting macrophage migration patterns revealed a population of cells that remain in the inter-wound region seemingly lost within this field of competing attractant cues and show no clear bias in their migration toward either wound (Figures 4C and 4E–4G). We then carried out the same experiment but instead generated one of the competing wounds in Mcr knockdown tissue and one in neighboring wild-type tissue. Analyzing the resulting migration patterns of the responding macrophages within the inter-wound region revealed that the macrophages occupying this region prior to wounding are no longer confused but, instead, migrate toward the wild-type wound, ignoring that made to the Mcr knockdown tissue (Figures 4D–4G). Consistent with our model, we sporadically observe instances of macrophages

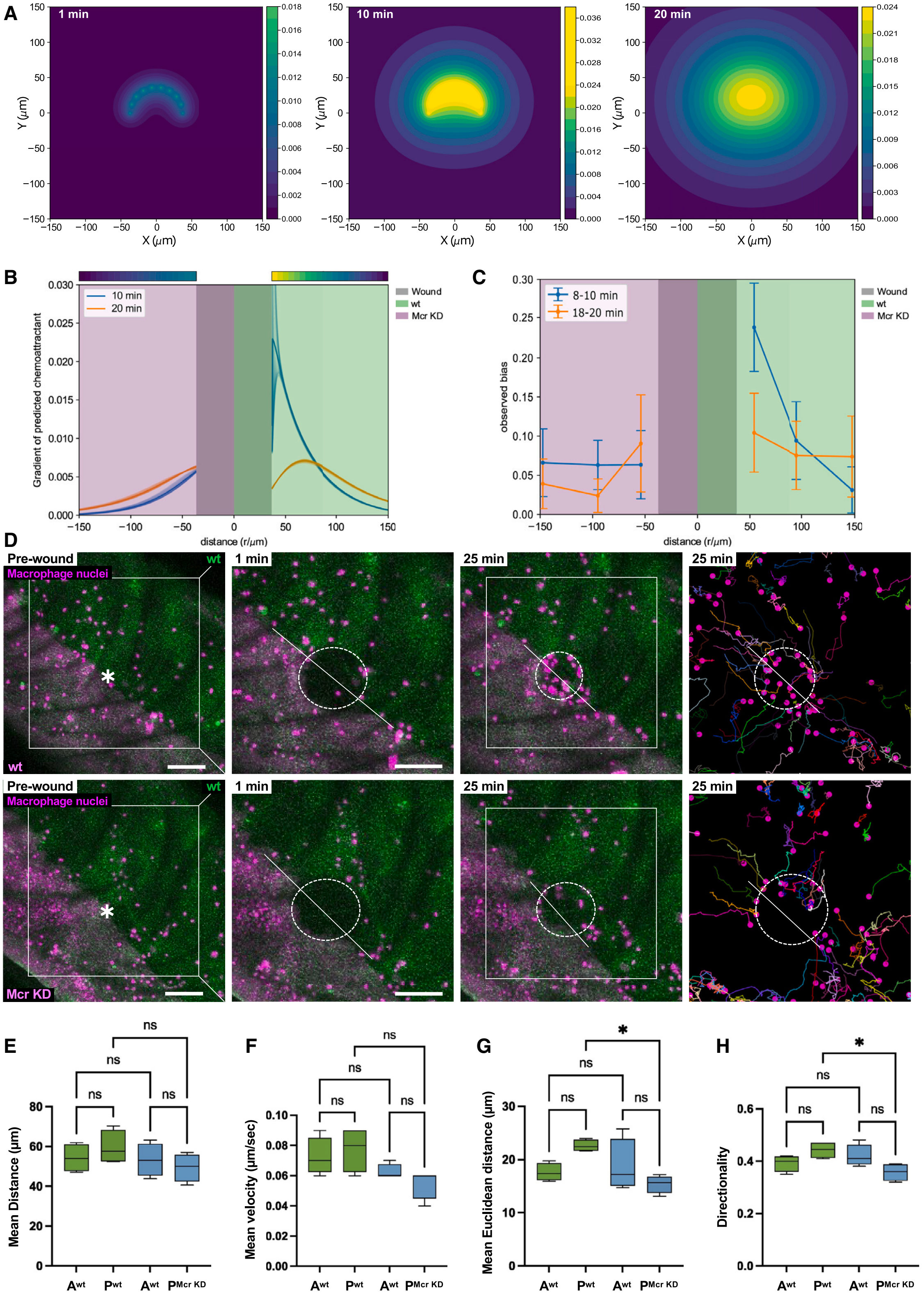

Figure 3. Macrophage behavior in the presence of restricted chemoattractant signal generation

(A) Snapshots of predicted chemoattractant distribution (in arbitrary units) at 1, 10, and 20 min post wounding (with $D = 200\mu m^2/min, \tau = 18$ min) approximating the "half wounds" as a semicircle of point sources along the wound edge.

(legend continued on next page)

that initially lie closer to the Mcr knockdown wound, re-orientate themselves, and migrate away from the mutant wound in favor of the wild-type site of damage, essentially behaving as if they were exposed to a single wound (Figure S5A). Finally, to demonstrate that Mcr works as a bona fide macrophage chemoattractant, we ectopically expressed Mcr, in the absence of a wound, by crossing UAS-Mcr to ems-Gal4, which drives specifically within the bottle cells of the developing spiracles of the *Drosophila* embryo.[16] Ectopic expression of Mcr within the bottle cells leads to a robust increase in macrophage number surrounding these structures (Figures 4H and 4I; Video S5).

Taken together, our results demonstrate a clear chemotactic role for Mcr in driving the rapid recruitment of inflammatory cells to sites of damage in the fly. Mcr therefore plays a crucial role in ensuring an efficient immune response to damage, revealing a new function for this protein. We can now integrate these new findings with previous work to propose a two-part signaling model that drives acute inflammatory cell migration to sites of tissue damage. Our findings suggest that injury triggers the release of Mcr from epithelial cells, along with the calcium-dependent production of reactive oxygen species (ROS), including H_2O_2. H_2O_2 diffuses rapidly away from the wound and, through activation of a previously reported Src-kinase-dependent signaling pathway, leads to the phosphorylation of the damage receptor Draper on the membrane of neighboring macrophages. This initial signal ''primes'' the macrophage to respond to the second, slowly diffusing, damage signal, which we propose is Mcr. However, the exact molecular mechanism of Mcr's release and whether it operates upstream of Draper to drive the rapid directional migration of the inflammatory cells toward the site of injury remains to be clarified (Figure S5B).

Our study reveals fascinating new parallels between the mammalian complement system and its *Drosophila* counterpart. It is becoming clear that the mammalian complement system has more diverse roles beyond its traditional role in facilitating pathogen clearance at sites of infection and that the complement cascade can be triggered in the absence of infection by detection of damaged or altered self.[17–19] Activation of the blood clotting protease thrombin, for example, can activate the key complement components C3 and C5, leading to release of C3a and C5a anaphylatoxin fragments, respectively.[20,21] C5a lacks the thioester motif necessary to bind to and opsonize pathogens, but its cleavage product C5b, together with C6, forms the basis of the formation of the membrane attack complex. Anaphylatoxin fragment C5a, on the other hand, operates as a robust chemoattractant for neutrophils, monocytes, and macrophages.[22] Interestingly, Mcr is the only member of the *Drosophila* complement-like family of TEP proteins that, like mammalian C5, lacks the thioester motif, making it unable to bind to and opsonize membranes.[23] As is observed for C5a, we show here that Mcr instead operates as a robust chemoattractant for inflammatory blood cells. Sequence comparison of human C5 with *Drosophila* TEPs 1–6 shows a C5a-like cysteine-rich region in Mcr/TEP6 that is absent in the other TEPs (Figure S5C). Like C5a, this region in Mcr/TEP6 is flanked by a basic amino acid residue-specific proprotein convertase (BAR-PC) sequence (RPRR in C5a and PRR in Mcr/TEP6) and a serine protease cleavage site. AlphaFold prediction of the structure of this C5a-like sequence in Mcr/TEP6 (Figure S5D) shows that the six cysteine residues are predicted to form three disulfide bridges, as seen in C5a but following a different topology that is akin to the cysteine-rich complement-type ligand-binding repeats of lipoprotein-related receptor proteins (LRPs, DALI analysis).

Mcr is expressed in the pupal epithelium and localizes to septate junctions,[11,24] positioning it perfectly for response to a loss of adhesion following epithelial damage. Whether its release from wounded epithelium requires proteolytic cleavage, as is the case for C5a, and what triggers that cleavage requires further investigation, but its function as a damage-induced chemoattractant provides the first evidence of a chemotactic role for an insect complement-like protein and identifies Mcr as the *Drosophila* functional equivalent of mammalian complement component C5a. This finding now places *Drosophila* as a powerful model system to study, not only the traditional role of the complement system in pathogen recognition/opsonization but also the biology underpinning damage-induced C5 complement activation—a critical regulator of sterile inflammation in human disease.

RESOURCE AVAILABILITY

Lead contact

Further information and requests for resources and reagents should be directed to and will be fulfilled by the lead contact, Will Wood (w.wood@ed.ac.uk).

Materials availability

This study did not generate new unique reagents. Fly strains used in this study are available from the lead contact upon request.

Data and code availability

- All data reported in this paper will be shared by the lead contact upon request.
- Codes are available at https://github.com/Schumacher-group/ImmuneCellMigrationAnalysis.

(B) Gradient of the predicted chemoattractant concentration, which is proportional to the observed bias in the chemoattractant production-diffusion model (Figure 2; STAR Methods). Background color indicates the Mcr KD (magenta) and control (green) compartments of the epithelium and wound area (gray). The gradient was calculated as the spatial derivative of the data shown in (A) along the radial direction between azimuths $\pi/4 < \phi < 3\pi/4$ for distance > 0 (green background) and $-\pi/4 > \phi > -3\pi/4$ for distance < 0 (magenta background). Solid lines show the average gradient across the azimuth range, shaded lines show the full range.
(C) Observed bias toward the wound (gray), calculated separately from cell tracks binned by azimuth as in (A) in the Mcr KD (magenta) and control (green) compartments of the epithelium.
(D) Confocal time-lapse microscopy of pupal wing 18 h APF and macrophage tracks (right) showing the behavior of macrophages in the presence of a wound generated across the antero-posterior boundary when both compartments are wild-type (upper) or after Mcr KD in the posterior compartment (lower). Dotted lines highlight the antero-posterior boundary across the wound. Scale bars, 50 μm.
(E–H) Mean distance (E), mean velocity (F), mean Euclidean distance (G), and directionality (H) of macrophages as in (D), across the anterior (A) and posterior (P) compartments. Data are represented as mean ± SEM. Asterisks indicate significant differences (*$p < 0.05$; ns, not significant).
See also Video S4 and Tables S1 and S2.

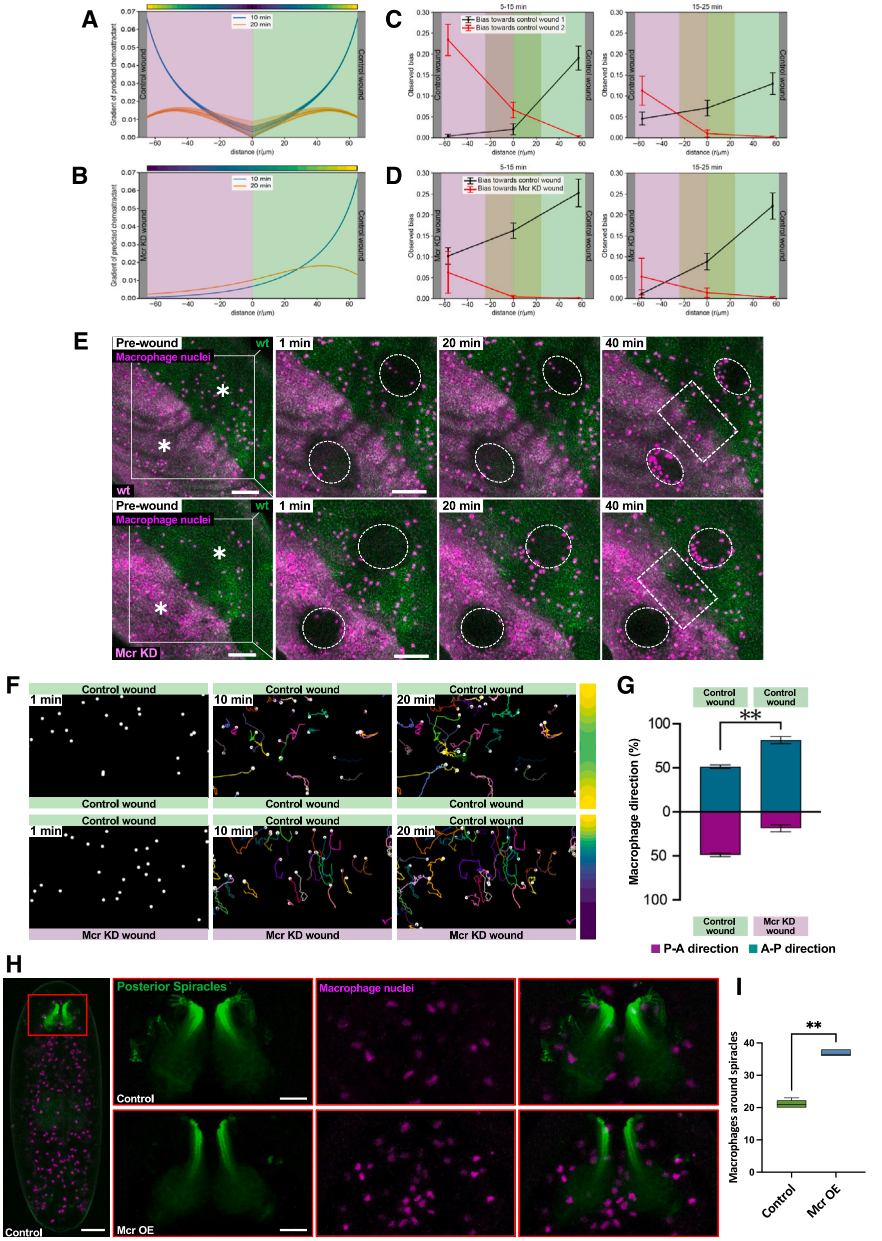

Figure 4. Macrophage behavior in the presence of two competing attractant signals

(A and B) Gradient of the predicted chemoattractant concentration between two wounds. Background color indicates the posterior (magenta) and anterior (green) sides of the tissue. The gradient was calculated as the spatial derivative of the data shown in snapshots along the radial direction from the center of either wound between azimuths $\pi/4 < \phi < 3\pi/4$ for distance < 0 (magenta background) and $-\pi/4 > \phi > -3\pi/4$ for distance > 0 (green background). Solid lines show the average gradient across the azimuth range, shaded lines show the full range.

(C and D) Observed bias toward two competing wounds (gray), calculated separately from cell tracks binned by azimuth as in (A); control vs. control wounds (upper), control vs. Mcr KD wound (lower); shaded area represents central region of highest chemoattractant overlap that lies at the boundary of posterior (magenta) and anterior (green) compartments of the epithelium.

(E) Time-lapse microscopy showing the behavior of macrophages in the presence of two competing wild-type wounds (upper) or a wild-type and a Mcr KD wound (lower) in the posterior compartment. Rectangles highlight the inter-wound regions where the chemoattractant signals overlap. Scale bars, 50 μm.

(F) Macrophage tracks in the inter-wound region at 1, 10, and 20 min post wounding when subjected to two wild-type wounds (upper) or in presence of a wild-type and an MCR KD wound (lower).

(G) Quantification of the macrophages in the inter-wound region toward the anterior and posterior compartment.

(H) Confocal microscope projection of stage 15 embryos (dorsal view), showing macrophage nuclei (dark magenta) surrounding the bottle cells of the posterior spiracles (green) in control conditions (upper) and following bottle-cell-specific Mcr overexpression (lower). Scale bars, 50 μm (left) and 20 μm (right).

(I) Quantification of the number of macrophages surrounding the spiracles.

Data in (G) and (I) are represented as mean ± SEM. Asterisks indicate significant differences (**$p < 0.01$).

See also Figures S4 and S5, Video S5, and Tables S1 and S2.

- Any additional information required to reanalyze the data reported in this paper is available from the lead contact upon request.

ACKNOWLEDGMENTS

We would like to thank FlyBase[25] as well as Bloomington Stock Center (University of Indiana, NIH P40OD018537) and the Vienna Drosophila Resource Center[26] for providing *Drosophila* lines. The study would not have been possible without the critical work of H. Falconer (University of Edinburgh). We would also like to thank all members of the Wood lab and the Edinburgh Cell Death Collective (ECDC) as well as Dr. J. Houston for insightful discussion. This work is funded by a Wellcome Trust Investigator Award to W.W. (22460/Z/21/Z) and an MRC Programme grant (MR/W019264/1) to W.W., S.J.J., and C.D.L., as well as an Academy of Medical Sciences/Wellcome Trust/Gvmnt DBEIS/BHF/Diabetes UK Springboard Award (SBF003/1170) to L.J.S. For the purpose of open access, the author has applied a Creative Commons Attribution (CC BY) license to any author-accepted manuscript version arising from this submission.

AUTHOR CONTRIBUTIONS

Conceptualization, W.W.; methodology, L.Z., A.S., A.J.D., D.R.T., L.J.S., and W.W.; formal analysis, L.Z., A.S., D.R.T., H.T., J.S.C., E.A., T.S., J.v.d.E., and L.J.S.; investigation, L.Z., A.S., T.S., D.R.T., H.T., J.S.C., J.v.d.E., and L.J.S.; writing – original draft, W.W., L.Z., A.S., and D.R.T.; writing – review & editing, W.W., L.Z., A.S., L.J.S., and J.v.d.E.; visualization, L.Z., A.S., and L.J.S.; funding acquisition, W.W., L.J.S., S.J.J., and C.D.L.

DECLARATION OF INTERESTS

The authors declare no competing interests.

CellPress
OPEN ACCESS

STAR★METHODS

Detailed methods are provided in the online version of this paper and include the following:

- KEY RESOURCES TABLE
- EXPERIMENTAL MODEL AND SUBJECT DETAILS
  - *Drosophila* maintenance and Genetics
- METHOD DETAILS
  - *Drosophila* pupal wounding
  - *Drosophila* embryo collection and mounting
  - Epithelial Wounding
  - Image acquisition and analysis
- QUANTIFICATION AND STATISTICAL ANALYSIS
  - Statistical analysis
  - Mathematical modeling
  - Chemoattractant production-diffusion model
  - Inference of chemoattractant production-diffusion parameters

SUPPLEMENTAL INFORMATION

Supplemental information can be found online at https://doi.org/10.1016/j.cub.2025.02.036.

Received: January 25, 2024
Revised: December 19, 2024
Accepted: February 18, 2025
Published: March 18, 2025

REFERENCES

1. Evans, I.R., and Wood, W. (2014). Drosophila blood cell chemotaxis. Curr. Opin. Cell Biol. *30*, 1–8. https://doi.org/10.1016/j.ceb.2014.04.002.
2. Moreira, S., Stramer, B., Evans, I., Wood, W., and Martin, P. (2010). Prioritization of competing damage and developmental signals by migrating macrophages in the Drosophila embryo. Curr. Biol. *20*, 464–470. https://doi.org/10.1016/j.cub.2010.01.047.
3. Wang, L., Kounatidis, I., and Ligoxygakis, P. (2014). Drosophila as a model to study the role of blood cells in inflammation, innate immunity and cancer. Front. Cell. Infect. Microbiol. *3*, 113. https://doi.org/10.3389/fcimb.2013.00113.
4. Ratheesh, A., Belyaeva, V., and Siekhaus, D.E. (2015). Drosophila immune cell migration and adhesion during embryonic development and larval immune responses. Curr. Opin. Cell Biol. *36*, 71–79. https://doi.org/10.1016/j.ceb.2015.07.003.
5. Stramer, B., Wood, W., Galko, M.J., Redd, M.J., Jacinto, A., Parkhurst, S.M., and Martin, P. (2005). Live imaging of wound inflammation in Drosophila embryos reveals key roles for small GTPases during in vivo cell migration. J. Cell Biol. *168*, 567–573. https://doi.org/10.1083/jcb.200405120.
6. Niethammer, P., Grabher, C., Look, A.T., and Mitchison, T.J. (2009). A tissue-scale gradient of hydrogen peroxide mediates rapid wound detection in zebrafish. Nature *459*, 996–999. https://doi.org/10.1038/nature08119.
7. Wood, W., and Martin, P. (2017). Macrophage functions in tissue patterning and disease: new insights from the fly. Dev. Cell *40*, 221–233. https://doi.org/10.1016/j.devcel.2017.01.001.
8. Weavers, H., Liepe, J., Sim, A., Wood, W., Martin, P., and Stumpf, M.P.H. (2016). Systems analysis of the dynamic inflammatory response to tissue damage reveals spatiotemporal properties of the wound attractant gradient. Curr. Biol. *26*, 1975–1989. https://doi.org/10.1016/j.cub.2016.06.012.
9. Evans, I.R., Rodrigues, F.S.L.M., Armitage, E.L., and Wood, W. (2015). Draper/CED-1 mediates an ancient damage response to control inflammatory blood cell migration in vivo. Curr. Biol. *25*, 1606–1612. https://doi.org/10.1016/j.cub.2015.04.037.
10. Lin, L., Rodrigues, F.S.L.M., Kary, C., Contet, A., Logan, M., Baxter, R.H.G., Wood, W., and Baehrecke, E.H. (2017). Complement-related regulates autophagy in neighboring cells. Cell *170*, 158–171.e8. https://doi.org/10.1016/j.cell.2017.06.018.
11. Bätz, T., Förster, D., and Luschnig, S. (2014). The transmembrane protein Macroglobulin complement-related is essential for septate junction formation and epithelial barrier function in Drosophila. Development *141*, 899–908. https://doi.org/10.1242/dev.102160.
12. DaCrema, D., Bhandari, R., Karanja, F., Yano, R., and Halme, A. (2021). Ecdysone regulates the Drosophila imaginal disc epithelial barrier, determining the length of regeneration checkpoint delay. Development *148*, dev195057. https://doi.org/10.1242/dev.195057.
13. Ponte, S., Jacinto, A., and Carvalho, L. (2019). The occluding junction protein Neurexin-IV is required for tissue integrity in the Drosophila wing disc epithelium. Matters *5*, 1–8.
14. Gupta, A.P., and Han, S.S. (1988). Arthropod immune system. III. Septate junctions in the hemocytic capsule of the german cockroach, Blattella germanica. Tissue Cell *20*, 629–634. https://doi.org/10.1016/0040-8166(88)90064-x.
15. Stroschein-Stevenson, S.L., Foley, E., O'Farrell, P.H., and Johnson, A.D. (2006). Identification of Drosophila gene products required for phagocytosis of Candida albicans. PLoS Biol. *4*, e4. https://doi.org/10.1371/journal.pbio.0040004.
16. Simões, S., Denholm, B., Azevedo, D., Sotillos, S., Martin, P., Skaer, H., Hombría, J.C.G., and Jacinto, A. (2006). Compartmentalisation of Rho regulators directs cell invagination during tissue morphogenesis. Development *133*, 4257–4267. https://doi.org/10.1242/dev.02588.
17. Ricklin, D., Hajishengallis, G., Yang, K., and Lambris, J.D. (2010). Complement: a key system for immune surveillance and homeostasis. Nat. Immunol. *11*, 785–797. https://doi.org/10.1038/ni.1923.
18. Pouw, R.B., and Ricklin, D. (2021). Tipping the balance: intricate roles of the complement system in disease and therapy. Semin. Immunopathol. *43*, 757–771. https://doi.org/10.1007/s00281-021-00892-7.
19. Lo, M.W., and Woodruff, T.M. (2020). Complement: Bridging the innate and adaptive immune systems in sterile inflammation. J. Leukoc. Biol. *108*, 339–351. https://doi.org/10.1002/JLB.3MIR0220-270R.
20. Huber-Lang, M., Sarma, J.V., Zetoune, F.S., Rittirsch, D., Neff, T.A., McGuire, S.R., Lambris, J.D., Warner, R.L., Flierl, M.A., Hoesel, L.M., et al. (2006). Generation of C5a in the absence of C3: a new complement activation pathway. Nat. Med. *12*, 682–687. https://doi.org/10.1038/nm1419.
21. Amara, U., Flierl, M.A., Rittirsch, D., Klos, A., Chen, H., Acker, B., Brückner, U.B., Nilsson, B., Gebhard, F., Lambris, J.D., et al. (2010). Molecular intercommunication between the complement and coagulation systems. J. Immunol. *185*, 5628–5636. https://doi.org/10.4049/jimmunol.0903678.
22. Guo, R.F., and Ward, P.A. (2005). Role of C5a in inflammatory responses. Annu. Rev. Immunol. *23*, 821–852. https://doi.org/10.1146/annurev.immunol.23.021704.115835.
23. Shokal, U., and Eleftherianos, I. (2017). Evolution and function of thioester-containing proteins and the complement system in the innate immune response. Front. Immunol. *8*, 759. https://doi.org/10.3389/fimmu.2017.00759.
24. Hall, S., Bone, C., Oshima, K., Zhang, L., McGraw, M., Lucas, B., Fehon, R.G., and Ward, R.E.t. (2014). Macroglobulin complement-related encodes a protein required for septate junction organization and paracellular barrier function in Drosophila. Development *141*, 889–898. https://doi.org/10.1242/dev.102152.
25. Marygold, S.J., Crosby, M.A., and Goodman, J.L.; FlyBase Consortium (2016). Using FlyBase, a database of Drosophila genes and genomes. Methods Mol. Biol. *1478*, 1–31. https://doi.org/10.1007/978-1-4939-6371-3_1.
26. Dietzl, G., Chen, D., Schnorrer, F., Su, K.C., Barinova, Y., Fellner, M., Gasser, B., Kinsey, K., Oppel, S., Scheiblauer, S., et al. (2007). A

genome-wide transgenic RNAi library for conditional gene inactivation in Drosophila. Nature *448*, 151–156. https://doi.org/10.1038/nature05954.

27. Millard, T.H., and Martin, P. (2008). Dynamic analysis of filopodial interactions during the zippering phase of Drosophila dorsal closure. Development *135*, 621–626. https://doi.org/10.1242/dev.014001.
28. Campbell, J.S., Davidson, A.J., Todd, H., Rodrigues, F.S.L.M., Elliot, A.M., Early, J.J., Lyons, D.A., Feng, Y., and Wood, W. (2021). PTPN21/Pez is a novel and evolutionarily conserved key regulator of inflammation in vivo. Curr. Biol. *31*, 875–883.e5. https://doi.org/10.1016/j.cub.2020.11.014.
29. Merabet, S., Catala, F., Pradel, J., and Graba, Y. (2002). A green fluorescent protein reporter genetic screen that identifies modifiers of Hox gene function in the Drosophila embryo. Genetics *162*, 189–202. https://doi.org/10.1093/genetics/162.1.189.
30. Schindelin, J., Arganda-Carreras, I., Frise, E., Kaynig, V., Longair, M., Pietzsch, T., Preibisch, S., Rueden, C., Saalfeld, S., Schmid, B., et al. (2012). Fiji: an open-source platform for biological-image analysis. Nat. Methods *9*, 676–682. https://doi.org/10.1038/nmeth.2019.
31. Ershov, D., Phan, M.S., Pylvänäinen, J.W., Rigaud, S.U., Le Blanc, L., Charles-Orszag, A., Conway, J.R.W., Laine, R.F., Roy, N.H., Bonazzi, D., et al. (2022). TrackMate 7: integrating state-of-the-art segmentation algorithms into tracking pipelines. Nat. Methods *19*, 829–832. https://doi.org/10.1038/s41592-022-01507-1.
32. Foreman-Mackey, D., Hogg, D.W., Lang, D., and Goodman, J. (2013). emcee: The MCMC Hammer. Publ. Astron. Soc. Pac. *125*, 306–312. https://doi.org/10.1086/670067.
33. Liepe, J., Taylor, H., Barnes, C.P., Huvet, M., Bugeon, L., Thorne, T., Lamb, J.R., Dallman, M.J., and Stumpf, M.P.H. (2012). Calibrating spatio-temporal models of leukocyte dynamics against in vivo live-imaging data using approximate Bayesian computation. Integr. Biol. (Camb.) *4*, 335–345. https://doi.org/10.1039/c2ib00175f.

STAR★METHODS

KEY RESOURCES TABLE

REAGENT or RESOURCE	SOURCE	IDENTIFIER
Deposited data		
Immune Cell Migration Analysis	This paper	GitHub: https://github.com/Schumacher-group/ImmuneCellMigrationAnalysis
Experimental models: Organisms/strains		
D. melanogaster: Ctr; w[1118]	Bloomington Drosophila Stock Center	RRID: BDSC_5905; FlyBase: FBal0018186
D. melanogaster: Mcr RNAi; P{KK104742}VIE-260B	Vienna Drosophila Resource Center	RRID: Flybase_FBst0457160
D. melanogaster: Srp3xcherry; w[1118] wg[Sp-1]/CyO; P{w[+mC]=srpHemo-3XmCherry}3/TM3, Ser[1]	Bloomington Drosophila Stock Center	RRID: BDSC_78359; FlyBase: FBti0197714
D. melanogaster: Srp-H2B-cherry; w[1118]; wg[Sp-1]/CyO; P{w[+mC]=srpHemo-H2A.3XmCherry}3	Bloomington Drosophila Stock Center	RRID: BDSC_78360; FlyBase: FBti0197716
D. melanogaster: ubi-DE-cadherin-GFP; w[*]; P{Ubi-p63E-shg.GFP}5	Kyoto Stock Center	RRID: DGGR_109007; FlyBase: FBst0307577
D. melanogaster: en-Gal4; y[1] w[*]; P{w[+mW.hs]=en2.4-GAL4}e22c/SM5	Bloomington Drosophila Stock Center	RRID: BDSC_1973 FlyBase: FBti0002970
D. melanogaster: uas-Moe-Cherry*; P{UASp-mCherry-Moe.ABD}*	Millard and Martin[27]	RRID: Flybase_FBtp0108918
D. melanogaster: *cora RNAi*; *y[1] sc[*] v[1] sev[21]; P{y[+t7.7] v[+t1.8]=TRiP.HMS01991}attP40*	Bloomington Drosophila Stock Center	RRID: BDSC_51845; FlyBase: FBti0157811
D. melanogaster: Nrx-IV RNAi; *y[1] v[1]; P{y[+t7.7] v[+t1.8]=TRiP.HMC03418}attP40*	Bloomington Drosophila Stock Center	RRID: BDSC_39071; FlyBase: FBti0149733
D. melanogaster: uas-GFP^{nls}; *w[1118]; P{w[+mC]=UAS-GFP.nls}14*	Bloomington Drosophila Stock Center	RRID: BDSC_4775; FlyBase: FBti0012492
D. melanogaster: Mcr^{EY07421}; y[1] w[67c23]; P{y[+mDint2] w[+mC]=EPgy2}Mcr[EY07421]/CyO	Bloomington Drosophila Stock Center	RRID: BDSC_15997; FlyBase: FBti0033377
D. melanogaster: Nrg^{GFP}; *w[1118] P{w[+mC]=PTT-GA}Nrg[G00305]*	Bloomington Drosophila Stock Center	RRID: BDSC_6844; FlyBase: FBti0027855
D. melanogaster: Srp-Gal4.2	Campbell et al.[28]	FlyBase: FBtp0147039
D. melanogaster: Ems-Gal4	Merabet et al.[29]	N/A
D. melanogaster: uas-Mcr	Eric Baehrecke, University of Massachusetts Medical School, USA	N/A
D. melanogaster: uas-GMA; *w[1118]; P{w[+mC]=UAS-GMA}2/SM6a*	Bloomington Drosophila Stock Center	RRID: BDSC_31775; FlyBase: FBti0131131
D. melanogaster: Srp-GMA	James Bloor, University of Kent, UK	N/A
Software and algorithms		
Zeiss Zen Black	https://www.zeiss.com/microscopy/int/products/microscope-software/zen.html	RRID: SCR_018163
NIH ImageJ/Fiji V2.3.0/1.53f	Marygold et al.[25]; https://imagej.net/	RRID: SCR_002285
Python 3.8.17	http://www.python.org/	RRID: SCR_008394
NumPy 1.20.3	http://www.numpy.org	RRID: SCR_008633
MatPlotLib 3.3.2	http://matplotlib.sourceforge.net/	RRID: SCR_008624
Pandas 0.25.3	https://pandas.pydata.org	RRID: SCR_018214
Skimage 0.16.2	https://github.com/Schumacher-group/ImmuneCellMigrationAnalysis	N/A

(*Continued on next page*)

Continued		
REAGENT or RESOURCE	SOURCE	IDENTIFIER
SciPy 1.3.3	http://www.scipy.org/	RRID: SCR_008058
tqdm 4.64.0	https://github.com/Schumacher-group/ImmuneCellMigrationAnalysis	N/A
Jupyter 4.4.0	https://github.com/Schumacher-group/ImmuneCellMigrationAnalysis	N/A
emcee 3.1.4	https://pypi.org/project/emcee/	N/A
GraphPad Prism V10	https://www.graphpad.com/scientific-software/prism/	RRID: SCR_002798
Adobe Illustrator 2024	https://www.adobe.com/uk/products/illustrator.html	RRID: SCR_010279
Inkscape 1.3.1	http://www.inkscape.org/	RRID: SCR_014479
HandBrake 1.7.2	https://handbrake.fr/	N/A
Microsoft Excel 16.81	https://www.microsoft.com/en-gb/	RRID: SCR_016137
Microsoft Word 16.81	https://www.microsoft.com/en-gb/	N/A
Microsoft Powerpoint 16.81	https://www.microsoft.com/en-gb/	RRID: SCR_023631
Endnote X9	http://endnote.com	RRID: SCR_014001
Chemicals, peptides, and recombinant proteins		
VOLTALEF oil 10 S	VWR	Cat# 24627.188
Other		
Nitrogen-Pumped Micropoint laser	Andor Technologies	N/A
Roe-Laser DPSL-Series	Rapp OptoElectronic GmbH	N/A
35 mm Dish-No. 0 Coverslip	MatTek	Cat# P35G-0-10-C
#5 Forceps	Dumonts	Cat# 11252-40
Cover Glasses No.1	VWR	Cat# 631-0133

EXPERIMENTAL MODEL AND SUBJECT DETAILS

Drosophila maintenance and Genetics

Drosophila stocks were kept in accordance with standard protocols, at 25°C with 50-60% relative humidity in a 12:12h light/dark cycle. Standard crosses were conducted to obtain the final experimental genotypes reported in Table S1. The F1 progeny of the desired genotype was selected by the presence of physical and fluorescent markers.

METHOD DETAILS

Drosophila pupal wounding

Pupae were allowed to reach the specified developmental stage (18 hours After Puparium Formation, APF) at 25°C. Pupae of the desired genotype were manually transferred to a piece of double-sided sticky tape affixed to a glass slide, securing the ventral side to the tape, and orienting the dorsal side upwards. Pupal case was carefully removed using fine forceps and adhered to glass-bottom culture dishes. Pupae were oriented to ensure most of the wing surface lays flat in direct contact with the glass. To prevent sample dehydration during imaging, a piece of absorbent filter paper soaked in distilled water was added to the side of the glass-bottomed dish, and the dish was covered with a lid.

Drosophila embryo collection and mounting

Embryos were collected on apple juice agar plates from overnight laying cages maintained at 25°C. The embryos were transferred to cell strainers, dechorionated in bleach for 90 seconds, and rinsed thoroughly with distilled water. Stage 15 embryos were manually selected and mounted dorsal side up on glass slides coated with double-sided sticky tape. The embryos were then embedded in VOLTALEF oil 10 S and covered with a No. 1.0 coverslip.

Epithelial Wounding

Epithelial wounds were induced using laser ablation with either a Nitrogen-Pumped Micropoint laser system (Andor Technologies) or a Roe-Laser DPSL-Series (Rapp OptoElectronic GmbH) following manufacturer instructions.

Image acquisition and analysis

Confocal imaging was performed on a Zeiss LSM880 laser scanning confocal microscope equipped with 25X/0.8 and 40x/1.3 oil immersion objectives. GFP and mCherry were excited at 488 and 561 nm respectively. Pupal wing time lapses were acquired within 1 min post-wounding at 2 μm intervals over a total depth of 48 μm (25 stacks) every 30 sec. For embryonic wounds, images were acquired at 1 μm intervals over a total depth of 11 μm (12 stacks) every 30 sec. Images of embryonic posterior spiracles were acquired at 1 μm intervals over a total depth of 49 μm (50 stacks). The acquired images were imported into Fiji[30] and processed as required.

Region of interest around the wounds are reported in Table S2. Macrophage nuclei were tracked using TrackMate 7[31] Fiji plugin, and the resulting tracks were analyzed with the Chemotaxis tool (ibidi GmbH) Fiji plugin to determine coordinates, velocity, distance and directionality values of macrophage populations. Macrophage nuclei were counted manually in Figures 4H and 4I.

Videos were converted in.mp4 format (Video codec: H.264) using HandBrake 1.7.2.

QUANTIFICATION AND STATISTICAL ANALYSIS

Statistical analysis

All datasets underwent Shapiro-Wilk normality tests to ensure that the appropriate statistical tests were performed where relevant. Two-tailed unpaired t-tests with Welch's correction or Mann-Whitney tests were then performed on normally distributed and non-normally distributed data respectively. ANOVA test was performed for multiple comparison. All statistical analysis were performed with GraphPad Prism 10. All graphs show Mean±SD.

Mathematical modeling

Image processing and cell tracking

All image processing and cell tracking was performed in ImageJ.[30] Firstly, a region of interest was created around the pupal wing, excluding any macrophages outside the wing area, or a restricted region of interest was manually selected around the wound for further processing as detailed in Table S2. Cells were tracked using the ImageJ plugin TrackMate,[31] using the Laplacian of Gaussian (Log) filter to detect cell nuclei and LAP tracker and filtering fro track quality. Manual review of the spot detection was applied when needed. Cell tracks from multiple pupae under the same experimental conditions were grouped for further analysis.

Two-step computational inference pipeline

Our computational inference pipeline in Python followed the methodology described previously,[8] reimplemented by us in open-source Python code, which utilizes two main steps:

1) Biased-persistent Random Walk Modeling.
2) Chemoattractant Production-Diffusion Modeling.

Bias-persistent random walk model. Within the bias-persistent random walk model, cells are treated as non-interacting particles moving in two dimensions (2D). As previously described,[8] cell motion is primarily characterized by the turning angle (γ_t), at time t, which is a stochastic variable. The distribution of the turning angle is modelled as a wrapped normal distribution:

$$N_w(\gamma_t|\mu,\sigma) = \frac{1}{\sigma\sqrt{2\pi}}\sum_{i=-\infty}^{\infty}\exp\left(-\frac{(\gamma_t - \mu+2\pi i)^2}{2\sigma^2}\right),$$

where μ is the mean turning angle and σ is the variance, which depends on either the bias or persistence parameters. Cells take biased steps with probability w and persistent steps with probability $1 - w$. For biased steps we define $\mu = \beta$ (the bias angle defining the direction to the wound, see Figure 2A) and for persistent steps $\mu = \gamma_{t-1}$. The variances are given by $\sigma_b{}^2 = -2\log(b)$ and $\sigma_p{}^2 = -2\log(p)$, with the bias and persistence parameters, b and p, having values between 0 and 1. A single cell trajectory is therefore calculated by drawing a series of turning angles, given the set of parameters w,b,p.

Inference of observed bias and persistence. From the cell tracking data we infer the parameters of the biased-persistent random walk using Bayesian inference. The likelihood function for the three parameters w,b,p can be calculated exactly, and we sample the posterior distributions using an ensemble Monte Carlo scheme as implemented in the emcee[32] package in Python. We choose the prior distributions for w,b,p to be Uniform between 0 to 1, in accordance previous analysis.[8] Each emcee run used 10 walkers and 10000 steps, with a burn-in of 3000 samples or twice the maximum auto-correlation time of MCMC traces across all parameters and spatiotemporal bins, whichever is greater. From the inferred parameters w,b,p we visualise the observed bias = $w\times b$ and observed persistence $= (1 - w) \times p$.

Chemoattractant production-diffusion model

The production-diffusion model assumes that attractant is produced at point source locations, $r = 0,$ at a constant rate q up until a time τ. The concentration of chemoattractant at a distance r and time t is then given by

$$A(\boldsymbol{r},t) = \int_0^{\min(\tau,t)} \frac{q}{\sqrt{4D(t-t')\pi}}\exp\left(-\frac{r^2}{4D(t-t')}\right)dt',$$

where D is the diffusion coefficient. Alternatively, we can write this using the exponential integral, Ei, which allows more efficient numerical computation in our Python implementation:

$$A(\boldsymbol{r},t) = \begin{cases} -\dfrac{q}{4\pi D} Ei\left(-\dfrac{r^2}{4Dt}\right), t < \tau \\ \dfrac{q}{4\pi D}\left(Ei\left(-\dfrac{r^2}{4D(t-\tau)}\right) - Ei\left(-\dfrac{r^2}{4Dt}\right)\right), t \geq \tau \end{cases}$$

To model how the cells sense the chemoattractant, we follow previous analysis[8] to describe the steady-state concentration of receptor-ligand complex as:

$$C(r,t) = \frac{1}{2}(\kappa_d + R_0 + A(r,t)) - \sqrt{\frac{1}{4}(\kappa_d + R_0 + A(r,t))^2 - R_0 A(r,t)},$$

where κ_d is the dissociation constant, and R_0 is the total concentration of receptors. For simplicity each cell is modelled to sense the local concentration at both its front and rear, in the direction to the wound. The observed bias is then assumed to be a linear function of the difference in complex concentration:

$$Observed\ bias = m[C(r - \Delta r, t) - C(r + \Delta r, t)] + b_0$$

where m is a scaling constant, Δr is average radius of the cell, and b_0 is the baseline observed bias.

This allows us to leverage inference to understand the following attractant and cell characteristics, q, D, τ, m, R_0, K_d and b_0.

Inference of chemoattractant production-diffusion parameters

From the variation of the inferred observed bias in time space we infer parameters $\theta = q, D, \tau, m, R_0, K_d$ and b_0 of the model for chemoattractant production-diffusion (and sensing). For each spatiotemporal bin we approximate the (previously calculated) posterior distribution of observed bias values as a normal distribution, which allows us to calculate the likelihood as:

$$L(\theta) = \prod_{i=1}^{T}\prod_{j=1}^{S} p(ob_{i,j}|\theta),$$

where T is the number of time bins, S is the number of spatial bins and $p(ob_{i,j}|\theta)$ is the probability density (calculated from the normal distribution for each bin) of observed bias values given by the chemoattractant production-diffusion model with parameters θ. As the movement of cells that have already reached the wound edge is no longer primarily driven by the chemoattractant signal, we excluded these for the purpose of infering chemoattractant parameters. For simplicity, we did this by skipping the first spatial bin (0-70 microns), based on an estimated wound radius of 40 microns plus a macrophage diameter of 30 microns, though other choices could also reasonably be made, such as omitting the closest cells to the (contracting) wound edge at each time-point, or omotting cells based on a sharp drop in persistence when they reach the wound, for example. Each emcee run used 40 walkers for a maximum of 1000000 steps, or until the estimated auto-correlation time of the reasonably inferable parameters $D, \tau,$ and b_0 had converged and the chain length reached at least 50 times this autocorrelation time. Afterwards we discarded a burn-in of twice the autocorrelation time of the MCMC chain. The parameters were initialized with the following priors, using posterior distributions of previously published work as priors where relevant:

Parameter	Prior	Reference
Flow rate, q	Uniform(0,3500)	Liepe et al.[33] have a 1D diffusion model with amplitude parameter Uniform(0,1000) over 1.5hrs or more, per unit length, so we estimate 1000/90mins times wound circumference (here max 100 microns diameter) to be the upper bound for q
Diffusivity, D	For WT tissue: TruncatedNormal(200, 50);<br>For MCR KD: Uniform(0,1000)	Weavers et al.[8] Figure 3C
Production time, τ	For WT tissue: TruncatedNormal(18, 3);<br>For MCR KD: Uniform(0,25)	Weavers et al.[8] Figure 3D
m	Uniform(0, 100)	N/A
R_0	Uniform(0, 10000)	Liepe et al.[33] Table 1
κ_d	Uniform(0, 10000)	Liepe et al.[33] Table 1
Baseline bias, b_0	TruncatedNormal(0.02, 0.02)	Weavers et al.[8] Figure 3E

Current Biology, Volume *35*

Supplemental Information

Drosophila complement-like Mcr acts as a wound-induced inflammatory chemoattractant

Luigi Zechini, Henry Todd, Thibaut Sanchez, Daniel R. Tudor, Jennie S. Campbell, Edward Antonian, Stephen J. Jenkins, Christopher D. Lucas, Andrew J. Davidson, Jean van den Elsen, Linus J. Schumacher, Alessandro Scopelliti, and Will Wood

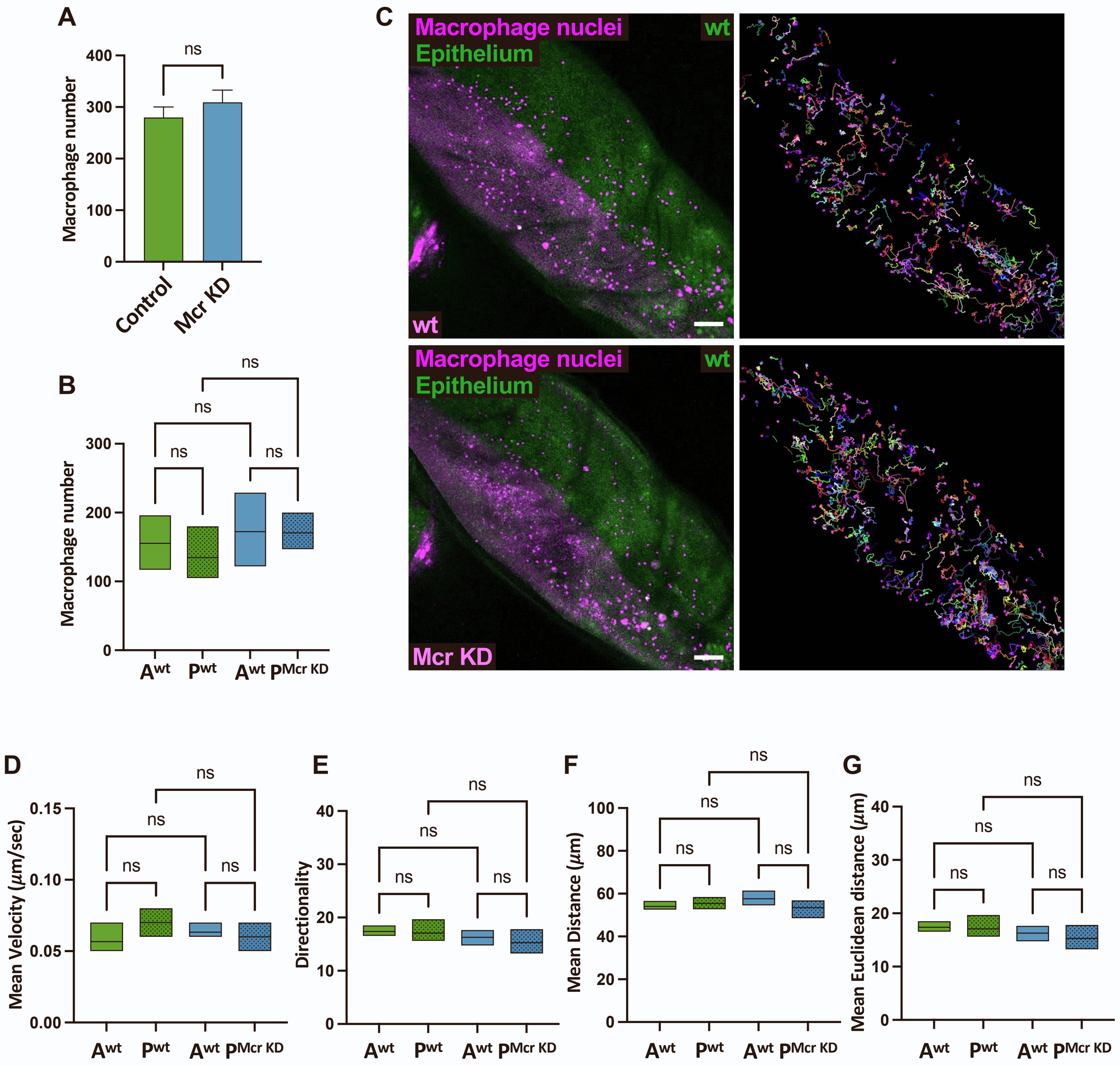
A
ns
Macrophage number
400
300
200
100
0
Control
Mcr KD
B
ns
ns
ns
ns
Macrophage number
300
200
100
0
A^wt
P^wt
A^wt
P^Mcr KD
C
Macrophage nuclei
Epithelium
wt
wt
Macrophage nuclei
Epithelium
wt
Mcr KD
D
ns
ns
ns
ns
Mean Velocity (μm/sec)
0.15
0.10
0.05
0.00
E
Directionality
40
30
20
10
0
F
Mean Distance (μm)
100
80
60
40
20
0
G
Mean Euclidean distance (μm)
40
30
20
10
0

Figure S1: Mcr KD in the posterior compartment of the pupal wing does not affect homeostatic macrophage behaviour. Related to Figure 1.

(A-B) Quantification of number of macrophages (A) and their distribution along the anterior and posterior compartments (B) in pupal wings 18h APF in control and upon Mcr KD in the posterior compartment, in the absence of a wound. **(C)** Representative frame of a time-lapse movie of macrophage nuclei (magenta) in an unwounded pupal wing 18h APF, and relative tracks (right panels) in control and upon Mcr KD in the posterior compartment. Scale bar 50 μm. green: epithelium **(D-G)** Quantification of macrophage mean velocity (D), Directionality (E), Mean distance (F) and Mean Euclidean distance (G) across the Anterior (A) and Posterior (P) compartments as in A.

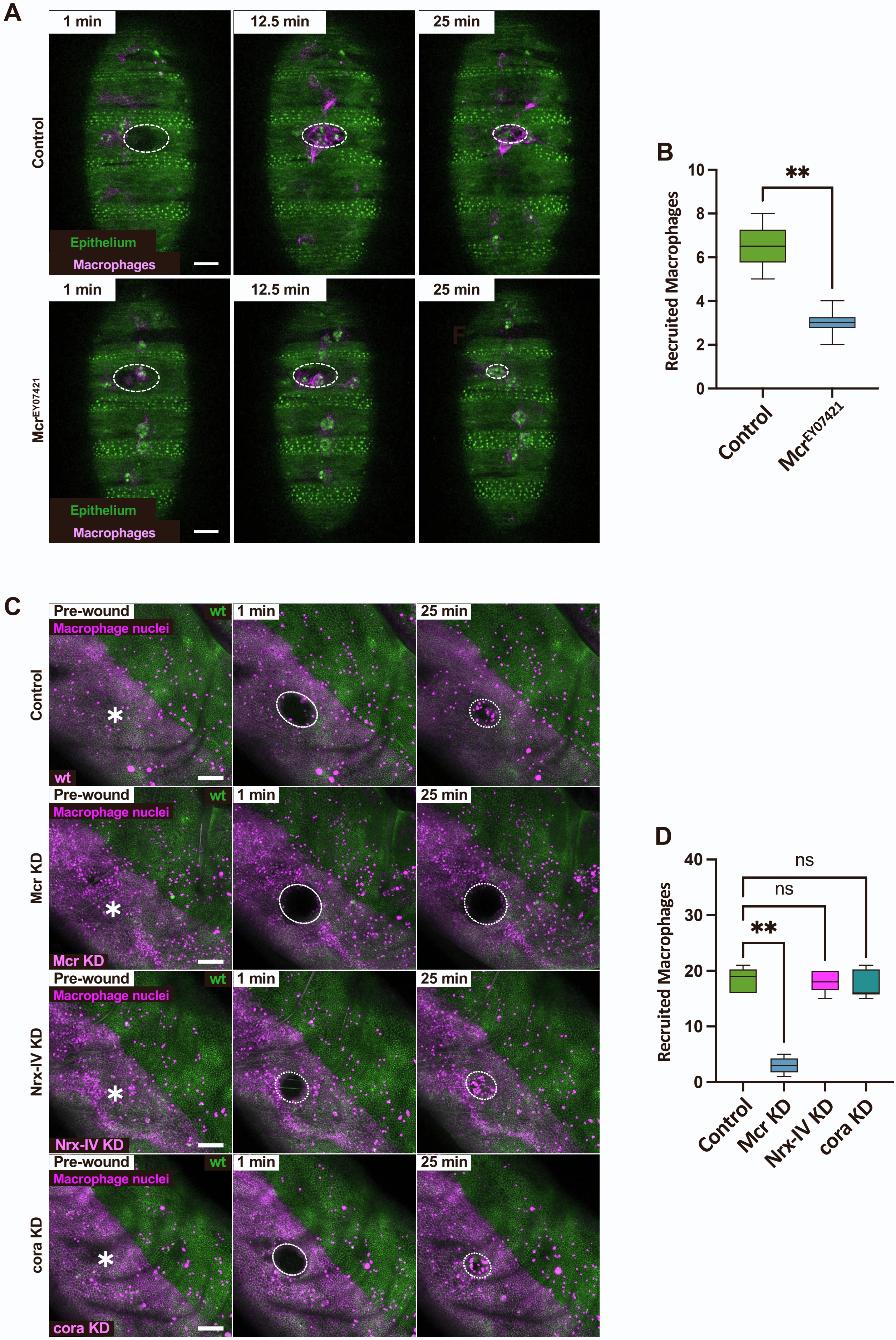
A
1 min
12.5 min
25 min
Control
Epithelium
Macrophages
McrEY07421
B
Recruited Macrophages
**
0
2
4
6
8
10
Control
McrEY07421
C
Pre-wound
wt
Macrophage nuclei
1 min
25 min
Control
Mcr KD
Nrx-IV KD
cora KD
D
Recruited Macrophages
0
10
20
30
40
ns
ns
**
Control
Mcr KD
Nrx-IV KD
cora KD

Figure S2: Epithelial Mcr is required for efficient macrophage recruitment to wounds independent of function in septate junctions. Related to Figure 1.

(A-B) Confocal time-lapse microscopy of wounded stage 15 control embryos and $Mcr^{EY07421}$ mutants (A) and quantification of macrophage number recruited to the wound site (B). **(C-D)** Confocal time-lapse microscopy of pupal wing 18h APF, showing macrophage nuclei (dark magenta dots) recruited to the wound site upon knockdown of the septate junction components Mcr, Nrx-IV and cora (C) and quantification of macrophage number recruited to the wound site (D).

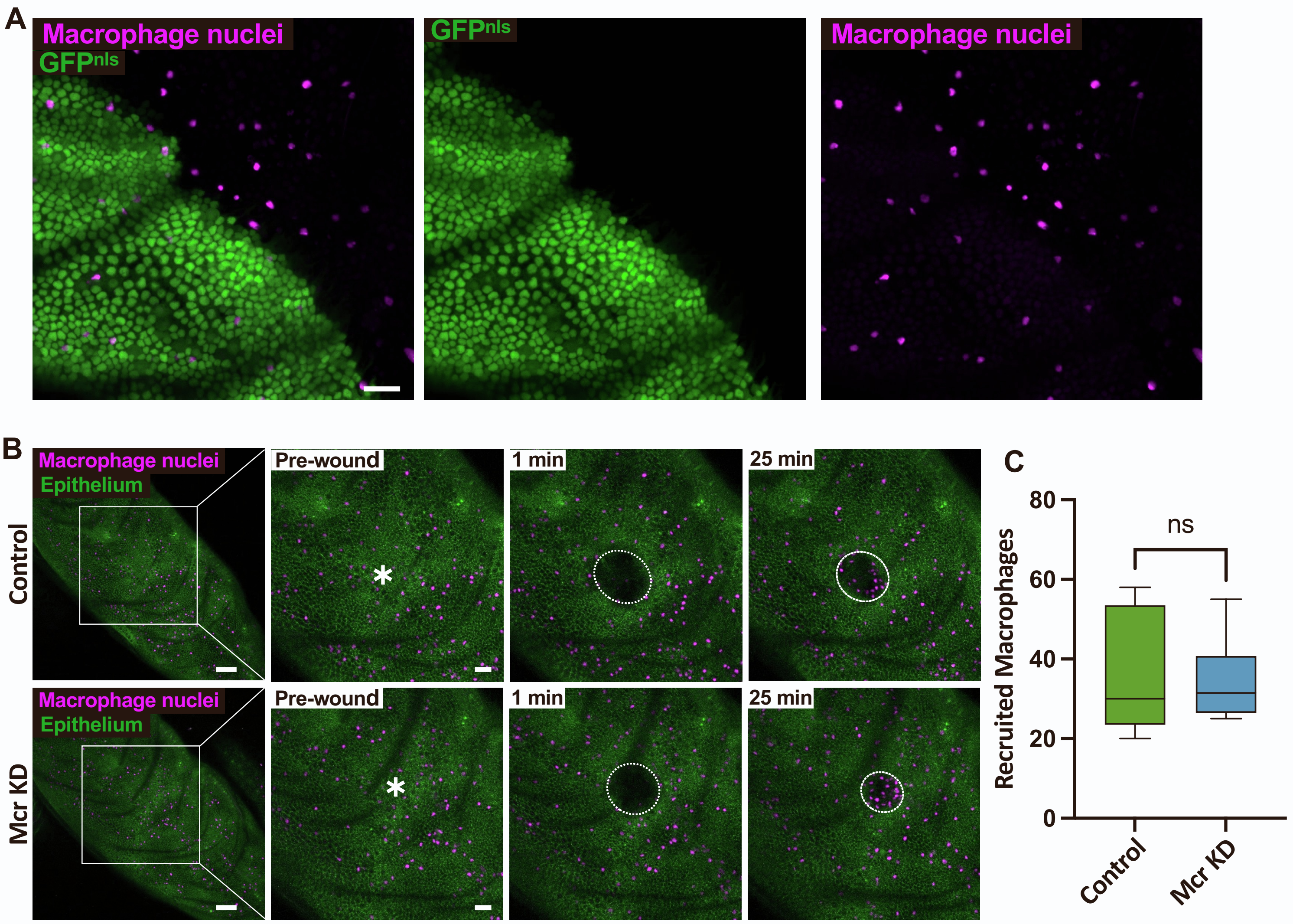
A
Macrophage nuclei
GFPnls
GFPnls
Macrophage nuclei
B
Macrophage nuclei
Epithelium
Control
Pre-wound
1 min
25 min
Macrophage nuclei
Epithelium
Mcr KD
Pre-wound
1 min
25 min
C
Recruited Macrophages
80
60
40
20
0
ns
Control
Mcr KD

Figure S3: Mcr KD within macrophages has no effect on wound recruitment. **Related to Figure 1.**

(A) Representative confocal projection showing the engrailed driver expressing a nuclear GFP (green) construct that has no overlap with the macrophage population (magenta). Scale bar 20 μm. **(B)** Time lapse imaging of macrophage recruitment to the wound at 1 and 25 min after wounding showing no defects in macrophage recruitment after macrophage specific Mcr KD. Scale bars: 50 μm (left panels), 20 μm (right panels); asterisks: wound location; green: epithelium. **(C)** Quantification of the macrophages recruited to the wound as in B.

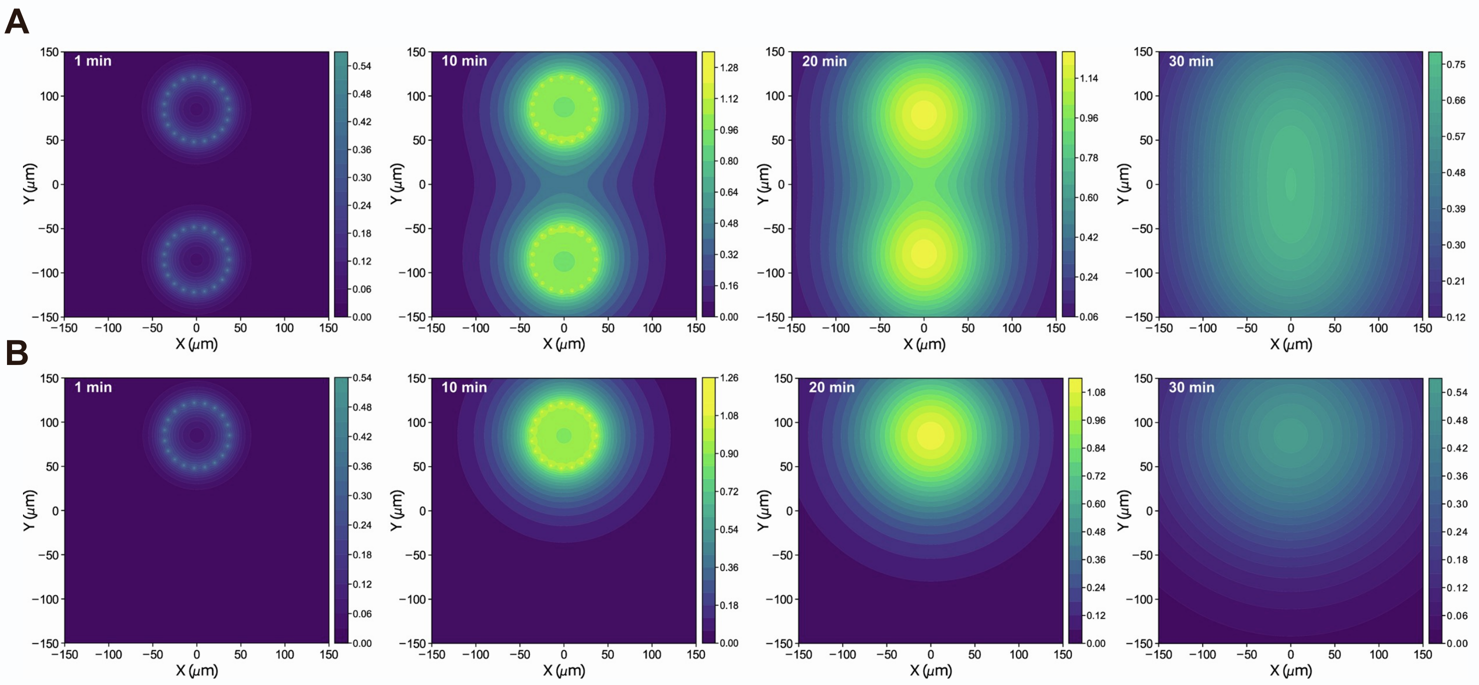
A
1 min
10 min
20 min
30 min
B
1 min
10 min
20 min
30 min
X (μm)
Y (μm)

Figure S4: Predicted chemoattractant distribution between two competing wounds. Related to Figure 4.

(A) Snapshots of predicted chemoattractant distribution (in arbitrary units) around and between two wounds at 1, 10, 20, and 30 min post wounding, using the same parameters as in Figure 4, and approximating the wounds as circles of point sources along the wound edge. **(B)** Snapshots of predicted chemoattractant distribution and gradient thereof, as in A but now with one Control wound and one lacking chemoattractant.

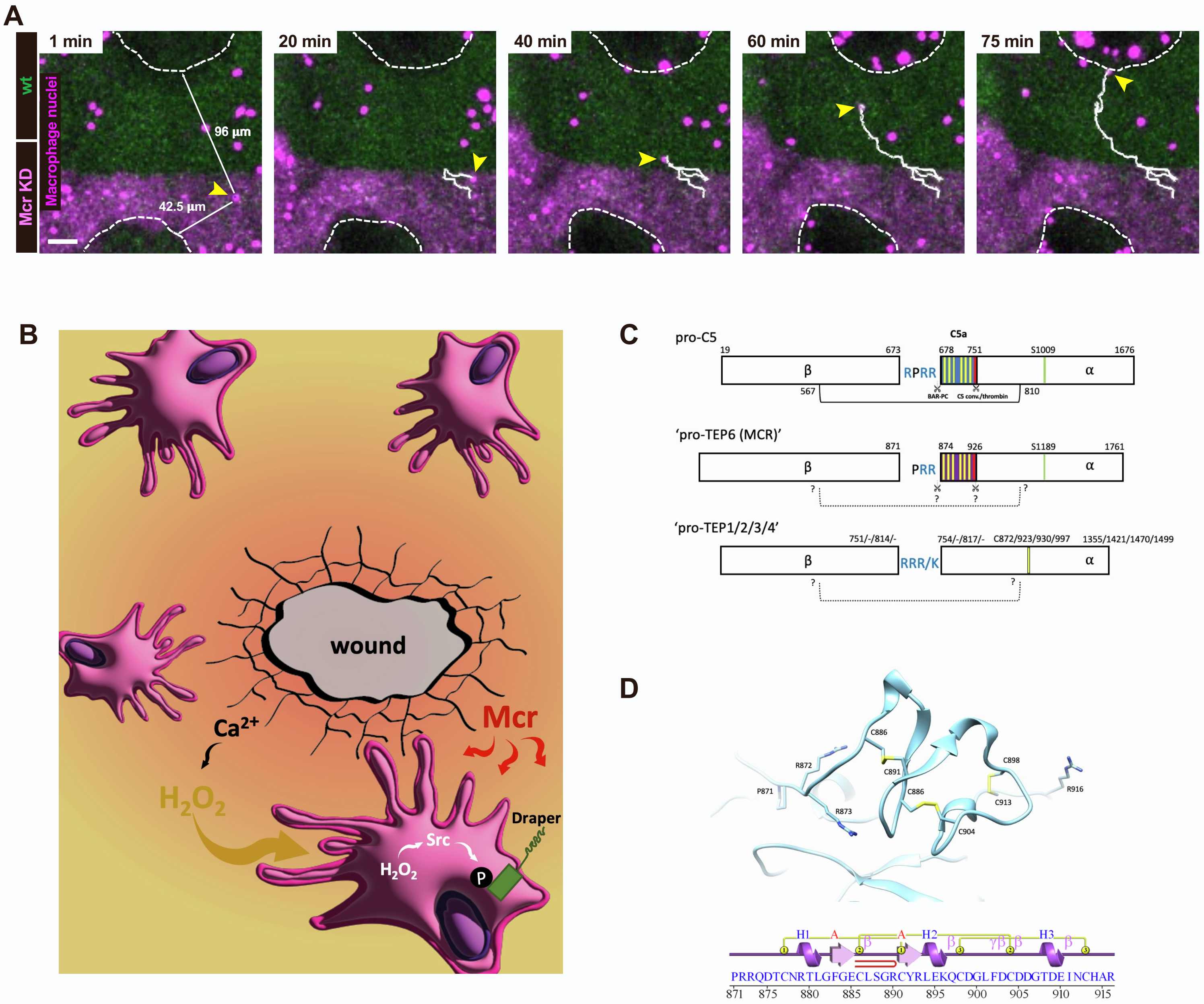
A
1 min
20 min
40 min
60 min
75 min
wt
Mcr KD
Macrophage nuclei
96 µm
42.5 µm
B
wound
Ca2+
H2O2
Mcr
Src
H2O2
P
Draper
C
pro-C5
C5a
19
673
678
751
S1009
1676
β
RPRR
α
567
BAR-PC
C5 conv./thrombin
810
'pro-TEP6 (MCR)'
871
874
926
S1189
1761
β
PRR
α
'pro-TEP1/2/3/4'
751/-/814/-
754/-/817/-
C872/923/930/997
1355/1421/1470/1499
β
RRR/K
α
D
C886
R872
C891
C898
P871
C886
R916
R873
C913
C904
H1
A
A
H2
H3
PRRQDTCNRTLGFGECLSGRCYRLEKQCDGLFDCDDGTDEINCHAR
871 875 880 885 890 895 900 905 910 915

Figure S5: Mcr operates as a macrophage chemoattractant *in vivo.* Related to Figure 4.

(A) Timelapse and overlap tracking (white line) of a single macrophage (yellow arrowhead) in close proximity to a wound generated in a Mcr-deficient epithelium that is attracted by a more distant wild-type wound. Scale bar 20 μm. **(B)** Schematic representation of the proposed role of Mcr (red) in coordinating with H_2O_2 to drive macrophage recruitment to wounds **(C)** Sequence comparison of human C5 with *Drosophila* TEPs 1-6 highlighting the presence of a C5a-like cysteine-rich region in Mcr/TEP6. **(D)** Alphafold prediction and topology diagram of the C5a-like sequence region in Mcr/TEP6. Shown are the positions of the potential basic amino acid residue-specific proprotein convertase (BAR-PC) cleavage sequence (PRR, upper panel) and serine protease cleavage site (R916, lower panel). The predicted structure of the MCR/TEP6 C5a-like region resembles the structure of cysteine-rich complement-type repeats seen in lipoprotein-related receptor proteins (DALI analysis), stapled together by three disulfide bonds. The ribbon diagram was created using UCSF Chimera and the topology image was generated using PDBsum (EMBL-EBI).

List of Genotypes		
1B-D	Control	; ubi-DE-cadherin-GFP, En-Gal4, uas-Moe-Cherry/+; Srp-3XmCherry/+
	Mcr KD	; ubi-DE-cadherin-GFP, En-Gal4, uas-Moe-Cherry/+; Srp-3XmCherry/ uas-Mcr RNAi
1E-M	Control	; ubi-DE-cadherin-GFP, En-Gal4, uas-Moe-Cherry/+; Srp-H2A-cherry/+
	Mcr KD	; ubi-DE-cadherin-GFP, En-Gal4, uas-Moe-Cherry/+; Srp-H2A-cherry/ uas-Mcr RNAi
2B-E	Control	; ubi-DE-cadherin-GFP, En-Gal4, uas-Moe-Cherry/+; Srp-H2A-cherry/+
	Mcr KD	; ubi-DE-cadherin-GFP, En-Gal4, uas-Moe-Cherry/+; Srp-H2A-cherry/ uas-Mcr RNAi
3C-H	Control	; ubi-DE-cadherin-GFP, En-Gal4, uas-Moe-Cherry/+; Srp-H2A-cherry/+
	Mcr KD	; ubi-DE-cadherin-GFP, En-Gal4, uas-Moe-Cherry/+; Srp-H2A-cherry/ uas-Mcr RNAi
4C-G	Control	; ubi-DE-cadherin-GFP, En-Gal4, uas-Moe-Cherry/+; Srp-H2A-cherry/+
	Mcr KD	; ubi-DE-cadherin-GFP, En-Gal4, uas-Moe-Cherry/+; Srp-H2A-cherry/ uas-Mcr RNAi
4H, I	Control	; EMS-Gal4, uas-GMA/+; Srp-H2A-cherry/+
	Mcr OE	; EMS-Gal4, uas-GMA/uas-Mcr; Srp-H2A-cherry/+
S1A-G	Control	; ubi-DE-cadherin-GFP, En-Gal4, uas-Moe-Cherry/+; Srp-H2A-cherry/+
	Mcr KD	; ubi-DE-cadherin-GFP, En-Gal4, uas-Moe-Cherry/+; Srp-H2A-cherry/ uas-Mcr RNAi
S2A, B	Control	w; e22c-Gal4, uas-Moe-Cherry, Srp-GMA; Srp-GMA
	$Mcr^{EY07421}$	w; e22c-Gal4, uas-Moe-Cherry, Srp-GMA, $Mcr^{EY07421}$; Srp-GMA
S2C, D	Control	; ubi-DE-cadherin-GFP, En-Gal4, uas-Moe-Cherry/+; Srp-H2A-cherry/+
	Mcr KD	; ubi-DE-cadherin-GFP, En-Gal4, uas-Moe-Cherry/+; Srp-H2A-cherry/ uas-Mcr RNAi
	Nrx-IV KD	; ubi-DE-cadherin-GFP, En-Gal4, uas-Moe-Cherry/Nrx-IV RNAi; Srp-H2A-cherry/ +
	cora KD	; ubi-DE-cadherin-GFP, En-Gal4, uas-Moe-Cherry/cora RNAi; Srp-H2A-cherry/ +
S3A		; En-Gal4, uas-GFP^{nls}/+; Srp-H2A-cherry/+
S3B, C	Control	Nrg^{GFP}; Srp-Gal4.2/+; Srp-H2A-cherry/+
	Mcr KD	Nrg^{GFP}; Srp-Gal4.2/+; Srp-H2A-cherry/uas-Mcr RNAi
S5A		; ubi-DE-cadherin-GFP, En-Gal4, uas-Moe-Cherry/+; Srp-H2A-cherry/ uas-Mcr RNAi

Table S1 - List of Genotypes. Related to figure 1-4 and S1-5.

Experimental details

Figure	Genotype	Sample	Macrophage number/tracks	ROI size
1B-D	Control	1	14	354μmx354μm
		2	22	
		3	15	
		4	18	
		5	16	
	Mcr KD	1	2	
		2	2	
		3	2	
		4	3	
		5	1	
1E-M	Control	1	62	150μmx150μm
		2	38	
		3	41	
		4	58	
		5	44	
	Mcr KD	1	62	
		2	67	
		3	42	
		4	51	
2B-E	Control	1	302	
		2	294	
		3	398	
		4	270	
		5	269	
		6	330	
	Mcr KD	1	432	
		2	315	
		3	318	
		4	317	
		5	329	
3C, D	Control	1	204	300μmx300μm
		2	181	
		3	184	
		4	159	
		5	242	
		6	202	
	Mcr KD	1	136	
		2	167	
		3	197	
		4	168	
		5	138	
		6	116	
3E-H	Control	1	A: 122 P: 90	300μmx300μm
		2	A: 87 P: 80	
		3	A: 118 P: 121	
		4	A: 103 P: 146	

	Mcr KD	1	A: 69 P: 79	
		2	A: 109 P: 78	
		3	A: 71 P: 92	
		4	A: 47 P: 76	
4C-E	Control	1	162	200μmx170μm
		2	146	
		3	123	
		4	178	
	Mcr KD	1	97	
		2	124	
		3	106	
		4	194	
4F, G	Control	1	24	147μmx85μm
		2	22	
		3	23	
		4	30	
	Mcr KD	1	25	
		2	20	
		3	34	
		4	34	
4H, I	Control	1	20	120μmx80μm
		2	22	
		3	23	
		4	21	
		5	20	
		6	21	
	Mcr OE	1	38	
		2	38	
		3	36	
		4	36	
		5	36	
		6	37	
S1A-G	Control	1	A: 196 P: 180	
		2	A: 153 P: 119	
		3	A: 117 P: 105	
	Mcr KD	1	A: 122 P: 147	
		2	A: 166 P: 165	
		3	A: 229 P: 200	
S2A, B	Control	1	6	
		2	8	
		3	7	
		4	6	
		5	7	
		6	5	
	$Mcr^{EY07421}$	1	2	
		2	3	
		3	3	

		4	4	
		5	3	
		6	3	
S2C, D	Control	1	20	
		2	16	
		3	20	
		4	16	
		5	21	
		6	18	
	Mcr KD	1	3	
		2	1	
		3	4	
		4	3	
		5	5	
		6	2	
	Nrx IR	1	20	
		2	20	
		3	17	
		4	15	
		5	17	
		6	19	
	cora IR	1	15	
		2	15	
		3	16	
		4	21	
		5	20	
		6	16	
S3B, C	Control	1	30	
		2	20	
		3	49	
		4	58	
		5	27	
	Mcr KD	1	25	
		2	36	
		3	55	
		4	34	
		5	29	
		6	27	

A: Anterior P: Posterior

Table S2 - Experimental details. Related to Figure 1-4 and S1-5.
